# Sulphur isotopes of alkaline magmas unlock long-term records of crustal recycling on Earth

**DOI:** 10.1038/s41467-019-12218-1

**Published:** 2019-09-16

**Authors:** William Hutchison, Rainer J. Babiel, Adrian A. Finch, Michael A. W. Marks, Gregor Markl, Adrian J. Boyce, Eva E. Stüeken, Henrik Friis, Anouk M. Borst, Nicola J. Horsburgh

**Affiliations:** 1 School of Earth and Environmental Sciences, University of St Andrews, St Andrews, KY16 9AL UK; 2 Mathematisch-Naturwissenschaftliche Fakultät, FB Geowissenschaften, Universität Tübingen, Wilhelmstrasse 56, 72074 Tübingen, Germany; 3 Scottish Universities Environmental Research Centre, Rankine Avenue, East Kilbride, G75 0QF UK; 4 Natural History Museum, University of Oslo, PO 1172 Blindern, 0318 Oslo, Norway

**Keywords:** Geochemistry, Geodynamics, Mineralogy

## Abstract

Earth’s surface and mantle sulphur reservoirs are connected via subduction, crustal recycling and volcanism. Although oceanic hotspot lavas currently provide the best constraints on the deep sulphur cycle, their restricted age range (<200 Ma) means they cannot reveal temporal variations in crustal recycling over Earth history. Sulphur-rich alkaline magmas offer the solution because they are associated with recycled sources (i.e. metasomatized lithospheric mantle and plumes) and, crucially, are found throughout the geological record. Here, we present a detailed study of sulphur isotope fractionation in a Mesoproterozoic alkaline province in Greenland and demonstrate that an enriched subduction-influenced source (δ^34^S of +1 to +5*‰)* can be reconstructed. A global δ^34^S compilation reveals secular variation in alkaline magma sources which support changes in the composition of the lithospheric mantle and/or Ga timescales for deep crustal recycling. Thus, alkaline magmas represent a powerful yet underutilized repository for interrogating crustal recycling through geological time.

## Abstract (web summary)

Sulphur isotopes track recycling of subducted crustal material, yet few igneous rocks preserve these signals over Earth history. Here, the authors investigate a billion-year-old alkaline province in Greenland and are able to reconstruct a recycled mantle source, thus alkaline rocks can be used to reveal crustal recycling through geological time.

## Introduction

Magmas are an integral component of Earth’s S cycle and are linked to the surface via subduction, mantle storage, crustal recycling and volcanism^1,2^. The most significant insights into the connections between the surface and mantle S reservoirs have been gained from studies of oceanic hotspots, particularly ocean island basalts (OIBs). A key observation is that several OIBs^2,3^ show mass independent S isotope fractionation, a feature characteristic of Archaean age sedimentary rocks^4^, which is usually expressed as Δ^33^S. These OIBs (Mangaia^2^ and Pitcairn^3^) are characterised by negative Δ^33^S and δ^34^S, which fingerprint Archaean crust in their mantle source. Importantly, other OIBs (Discovery^5^, Samoa^6^ and Canary Islands^7^) indicate post-Archaean S (Δ^33^S ≈ 0) with positive δ^34^S (~3‰) and are suggested to represent subducted Proterozoic sediments and/or serpentinized oceanic peridotites. Isotopic variations between different OIBs suggest that there are chemically distinct reservoirs of subducted crust within the mantle^8^ and that plumes sample this ancient crust and return it to the surface.

Despite these advances we do not fully understand how changing subducted S input (from sedimentary records) ties to mantle S output (from igneous records). Although marine sedimentary rocks reveal large S isotopic variations through geological time^9,10^ there are no comparable igneous time-series; this greatly limits our ability to quantify rates and timescales of surface S recycling. A key issue is that the oceanic record only extends to ~200 Ma; thus, OIBs cannot reveal temporal variations in crustal recycling over Earth history.

Alkaline magmas (silicate rocks and carbonatites) represent low-degree melts of volatile-rich mantle sources^11^. Their trace element signatures and radiogenic isotopes are similar to OIBs^12^ and they are often linked to recycled crustal materials^13–17^. While predominantly found on the continents, there are a number of oceanic alkaline localities related to OIBs (including the Canary Islands). In all cases, alkaline magma sources are associated with mantle plumes^18–20^ and/or sub-continental lithospheric mantle (SCLM)^21–23^ that has been metasomatised by fluids and melts derived from previously subducted slabs.

The advantage of alkaline rocks, compared to more common basaltic or granitic suites, is that their low viscosities, densities and temperatures promote rapid rise to the surface and they generally show limited evidence of crustal contamination^11,24^. Evolved alkaline rocks are also rich in S-bearing minerals^25^, which reflect the high solubility of S in carbonatitic^26^ and alkaline silicate^27^ melts. Carbonatites, for example, have average S concentrations of ~6000 ppm^28^, much greater than other terrestrial magmas erupted through continental crust (granites and degassed basaltic lavas typically have concentrations <100 ppm^1,29^). In short, because alkaline magmas are S-rich, found throughout the geological record, and genetically linked to previously subducted crust, they are potentially well suited for understanding S cycling between the surface and mantle.

**Fig. 1:**
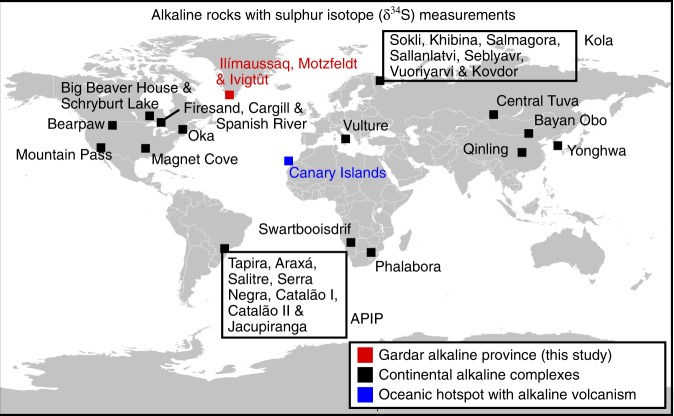
Locations of the alkaline rocks compiled for this study. The Kola Alkaline Province and the Alto Paranaiba Igneous Province (APIP), discussed in the text, are among the largest alkaline provinces known. Alkaline rocks are mostly found in continental settings although there are a few oceanic occurrences, including the Canary Islands hotspot

Before we can use S isotopes to investigate the origins of an igneous rock suite, we must account for all processes that may fractionate S from mantle source to surface. While there have been a large number of S isotope investigations of alkaline complexes (Fig. 1), few^30–33^ have thoroughly investigated how crustal contamination^34^, degassing^35^ and fluid evolution^36^ (i.e., changes in temperature-pH-fO_2_) altered the primary mantle signature. Understanding these processes is critical to unlocking the alkaline record of magma sources and placing these observations within the context of the global S cycle.

Here, we present a detailed S isotope study of an alkaline igneous province known as the Gardar (Fig. 1). We target primitive dykes and diatremes as well as three well-studied chemically evolved alkaline bodies (Ilímaussaq, Motzfeldt and Ivigtût, Fig. 1). By carefully screening for crustal contamination and interrogating degassing and magmatic fluid evolution processes we demonstrate that the δ^34^S of alkaline rocks can be used to evaluate mantle S sources. We undertake a global compilation and establish a δ^34^S time-series of alkaline magma sources. We show that most complexes require a component of recycled surface S, and find new evidence for a secular variation in their δ^34^S. We consider the causes of this temporal variation and demonstrate that alkaline rocks are a powerful data set for understanding connections between surface and mantle S reservoirs over geological timescales.

## Results

### Geological setting and sample selection

The Gardar province in SW-Greenland (Fig. 1) is a failed Mesoproterozoic continental rift that was volcanically and tectonically active in two cycles^37^ from 1320–1260 Ma and 1180–1140 Ma. Magmas were emplaced across and along the boundary between the North Atlantic Craton (>2800 Ma Archaean orthogneiss) and the Paleoproterozoic mobile belt (~1800 Ma Ketilidian granites, Supplementary Fig. 1). While the vast majority of Gardar magmas comprise intrusive suites (i.e., dyke swarms and km-scale plutonic bodies), extrusive lavas and tuffs are preserved as a package of rift-fill sediments known as the Eriksfjord Formation.

Geochemical investigations of Gardar rocks emphasise that their parental magmas were derived from a metasomatised SCLM^15,22,38^. Metasomatism has been linked to an episode of Andean-style subduction that took place during the Ketilidian orogeny and is supported by Gardar Nd-model ages, which mostly range between 1850 and 1720 Ma^22,39,40^, overlapping the ages of Ketilidian rocks. Thus, there is consensus that the Ketilidian orogeny led to pervasive mantle metasomatism; hence, Gardar magmas carry a geochemical signature of previously subducted crust and fluids^22,37,38^.

In this study, we analyse ENE-WSW oriented mafic dykes (commonly referred to as Giant Dykes), as well as lamprophyre and carbonatite diatremes. Previous investigations of these rocks^38,41^ confirm that they have been modified little by crustal interactions and therefore provide the best information on the Gardar mantle source^41^. We also investigate three intrusions: Ilímaussaq, Motzfeldt and Ivigtût (Fig. 1), which comprise alkaline and peralkaline (i.e., molar (Na + K)/Al > 1) igneous rocks of syenitic or alkali granitic affinity. While these intrusions are more chemically evolved than the dyke and diatreme samples they provide a valuable counterpoint to understand how magmatic and fluid evolution may impact S isotope systematics.

The geological history of the three alkaline intrusions is well-constrained and their magmatic evolution is markedly different^42,43^. In brief, Ilímaussaq hosts agpaitic nepheline syenites (peralkaline rocks with complex Na-Ca-HFSE minerals) and is one of the most reduced and chemically evolved magmatic series known^43^. Motzfeldt predominantly hosts miaskitic nepheline syenites (peralkaline rocks with zircon and Fe-Ti oxides), which formed at reduced magmatic conditions (less extreme than Ilímaussaq, but still below the QFM buffer^40^) and underwent intense late-stage oxidation^44^. Ivigtût is an alkaline granite stock that is associated with the world’s largest deposit of cryolite (Na_3_AlF_6_). The cryolite and its associated mineralisation represent the products of interaction between a magmatic fluid (dominated by CO_3_^2−^ and F^−^) and the host granite^42,45^.

Importantly, although reduced conditions (≤QFM) and high temperatures (1000–600 °C) mark the early magmatic phase of each complex, oxidised conditions (~HM) and lower temperatures (≤300 °C) prevail during later hydrothermal phases. Evidence for this transition includes haematite-rich skarns^46^, as well as fluid inclusion and mineral isotope studies (δD and δ^18^O) that support influx of external oxidising meteoric fluids and brines in the roof and margins of the complexes^14,40^.

### Sulphur isotope variations in Gardar rocks

**Fig. 2:**
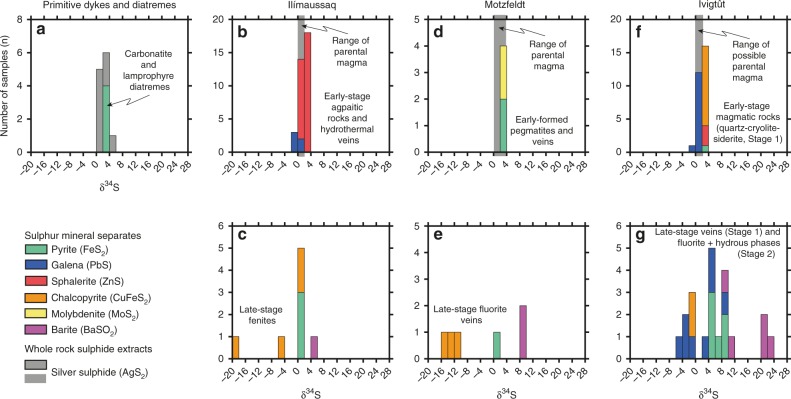
Histograms summarising sulphur isotope results for the Gardar. The bar colour corresponds to the sulphide or sulphate mineral analysed. Note that when sulphide minerals were finely disseminated we undertook a whole-rock sulphide extraction (converting to an Ag_2_S precipitate, see Methods). δ^34^S results from primitive mafic dykes are shown in panel **a** alongside δ^34^S analyses of pyrite extracted from carbonatite and lamprophyre diatremes. δ^34^S analyses of three alkaline complexes (Ilímaussaq, Motzfeldt and Ivigtût) are shown in **b**–**g**, with the upper panels **b**, **d** and **f** corresponding to early-formed magmatic rocks and veins, and the lower panels **c**, **e** and **g** corresponding to latest veins and fenites. Note that fenites are country rock around the intrusion that has been metasomatised by alkaline magmatic fluids. Note that in **b**, **d** and **f** the grey bar indicates the δ^34^S range of magmatic rocks and cumulate that are likely parental to the mineral separates analysed. All δ^34^S results are provided in Supplementary Data 1–2

δ^34^S analyses of Gardar rocks were carried out on mineral separates and whole-rock powders. For the latter, we undertook S concentration measurements and converted sulphides to Ag_2_S before isotopic analysis^47^ (Methods). Primitive dykes have whole-rock δ^34^S of 1–5 ‰ while pyrites from lamprophyre and carbonatite diatremes have δ^34^S of 2–3‰ (Fig. 2a). Gardar intrusions (Fig. 2b–g) show a greater δ^34^S span and are divided into early-formed magmatic rocks and veins, and late-stage veins and fenite (i.e., metasomatically altered country rock at the margins of the complex).

At Ilímaussaq, early-formed agpaitic rocks and hydrothermal veins (Fig. 2b) are dominated by sphalerite (1.3–3.1‰), with co-existing galena (–1.1 to 0.5‰) in a few samples. Syenitic rocks, cumulate and alkaline dykes that are parental to the agpaitic rocks and veins also show a narrow δ^34^S range (0.8–2.0‰, indicated by the grey bar in Fig. 2b). In contrast, late-stage fenite in the roof and margins of the intrusion contain sulphides (pyrite and chalcopyrite) with low and highly variable δ^34^S and sulphate (barite) with δ^34^S of 5.3‰ (Fig. 2c).

At Motzfeldt, we found molybdenite and pyrite in early-formed roof-zone pegmatites and veins with δ^34^S of 2.6–3.9‰ (Fig. 2d). Like Ilímaussaq, syenitic cumulate and veins from deeper in the intrusion show a similar δ^34^S range (1.3–3.4‰) that overlaps with the early-formed units (Fig. 2d). Late-stage fluorite veins injected into the surrounding rock (Fig. 2e) show variable δ^34^S with chalcopyrite down to –15‰ and barite up to 9‰.

At Ivigtût, sulphides from the early-formed quartz-cryolite-siderite magmatic body show a tight clustering of δ^34^S values (–1 to 3‰, Fig. 2f). Although these magmatic fluids were injected into an alkaline granite stock^42^, it is uncertain whether the granite is genetically related to the overlying cryolite body^45^. Nevertheless, granophyre dykes radiating from the deep granite reveal comparable δ^34^S values of 1‰ (Fig. 2f). Low-sulphide δ^34^S, down to –4.5‰, was observed in a few late-stage interstitial veins in cryolite, while high-sulphide δ^34^S, up to 9.4‰, was observed in the final alteration products that are rich in fluorite and hydrous phases (Fig. 2g). Late-stage barites also show two distinct values of ~10 and ~21‰.

### Magmatic processes that impart sulphur isotope variations

Whole-rock δ^34^S represent the total isotope value (δ^34^S_∑S_) of the melt or magmatic cumulate (depending on the sample) and will mainly be affected by crustal assimilation, magmatic degassing and melt-sulphide segregation^35^. Mineral δ^34^S record the isotopic fractionation between the S mineral phase and the melt or fluid. For an individual S-bearing mineral, the measured δ^34^S reflects the δ^34^S_∑S_ but also the temperature and S speciation of the melt/fluid (the latter being controlled by pH and fO_2_ conditions^36^). Thus, S minerals record δ^34^S variations due to changes in temperature-pH-fO_2_, features that are masked by a whole-rock approach. We first evaluate processes that impact δ^34^S_∑S_, and then assess changes in temperature-pH-fO_2_ encoded in mineral δ^34^S.

Table 1 compares S concentration and δ^34^S in local crust with Gardar magmas. Local crust is mostly magmatic in origin (i.e., orthogneiss, granites and rift-related lavas) with low S concentrations (<100 ppm) and a restricted δ^34^S range (1–4‰). Eriksfjord sediments have high-δ^34^S (25‰, consistent with a marine origin) but minimal S concentrations (~10 ppm) and we stress that there is no evidence for evaporitic units or shales with high S contents. Scenarios of crustal assimilation (melting and incorporating 10, 25 and 50% of local crust, Table 1) show that changes in melt δ^34^S for the magmatic suites are very low, generally <0.5‰. Calculations for Motzfeldt suggest that extreme crustal assimilation may have increased melt δ^34^S by ~1‰. However, it is important to note that the coarsely crystalline roof-zone pegmatites sampled for early-stage sulphide minerals (Fig. 2d) were not analysed for S concentrations (due to the difficulty of obtaining a representative whole-rock sample). As these samples are rich in visible sulphide minerals we expect S concentrations to be comparable to the agpaitic rocks of Ilímaussaq (~1000 ppm), i.e., much greater than the values used for modelling in Table 1. Hence, Motzfeldt magmas are unlikely to have been significantly shifted by crustal assimilation. Gardar magmatic suites show scant geochemical and petrographic evidence for crustal assimilation^38,40,43^; even under the most extreme scenarios (far greater than observed in modern continental rifts^48^) we find that crustal S concentrations are sufficiently low to discount this process.

**Table 1:** The impact of crustal contamination on sulphur isotopes

Magmatic suite	Average S (range), ppm	Average δ^34^S, ‰	Local crust	Average S (range), ppm	Average δ^34^S, ‰	δ^34^S Change due to bulk crustal assimilation, ‰		
						10%	25%	50%
Ilímaussaq	1430 (246–4413)	1.4	Eriksfjord sandstones	6	25.1†	0.01	0.03	0.05
			Eriksfjord mafic lavas	63 (38–79)	0.6	0.00	0.00	0.00
			Ketilidian granites	52 (12–76)	2.7	0.01	0.02	0.05
Motzfeldt	150 (100–200)	2.4	Eriksfjord sandstones	11	25.1	0.18	0.46	0.92
			Eriksfjord mafic lavas	37 (31–42)	1.0	0.03	0.06	0.13
			Ketilidian granites	25 (19–34)	4.4	0.07	0.18	0.36
Ivigtût	1000 (670–8100)	0.9*	Archaean gneiss	51 (31–99)	1.0	0.03	0.08	0.17
Regional dykes and diatremes	500 (357–772)	2.3	Eriksfjord sandstones	6	25.1†	0.03	0.08	0.15
			Eriksfjord mafic lavas	63 (38–79)	0.6	0.00	0.00	0.00
			Ketilidian granites	52 (12–76)	2.7	0.03	0.07	0.14

S concentration measurements and δ^34^S analyses of Gardar magmas and local crust are shown for whole-rock samples only (Supplementary Data 2). At Ivigtût whole-rock δ^34^S analyses (*) were only carried out on granophyre dykes radiating from the granite stock that underlies the main cryolite body. Note also that Eriksfjord sandstones labelled with † yielded insufficient Ag_2_S for isotopic analysis and a δ^34^S value of similar sandstones from Motzfeldt was used in calculations. The alkaline magmatic suites are significantly richer in S than the local crust (by 1–3 orders of magnitude). Thus, models of bulk crustal assimilation generate little variation in melt δ^34^S (generally, <0.5‰)

**Fig. 3:**
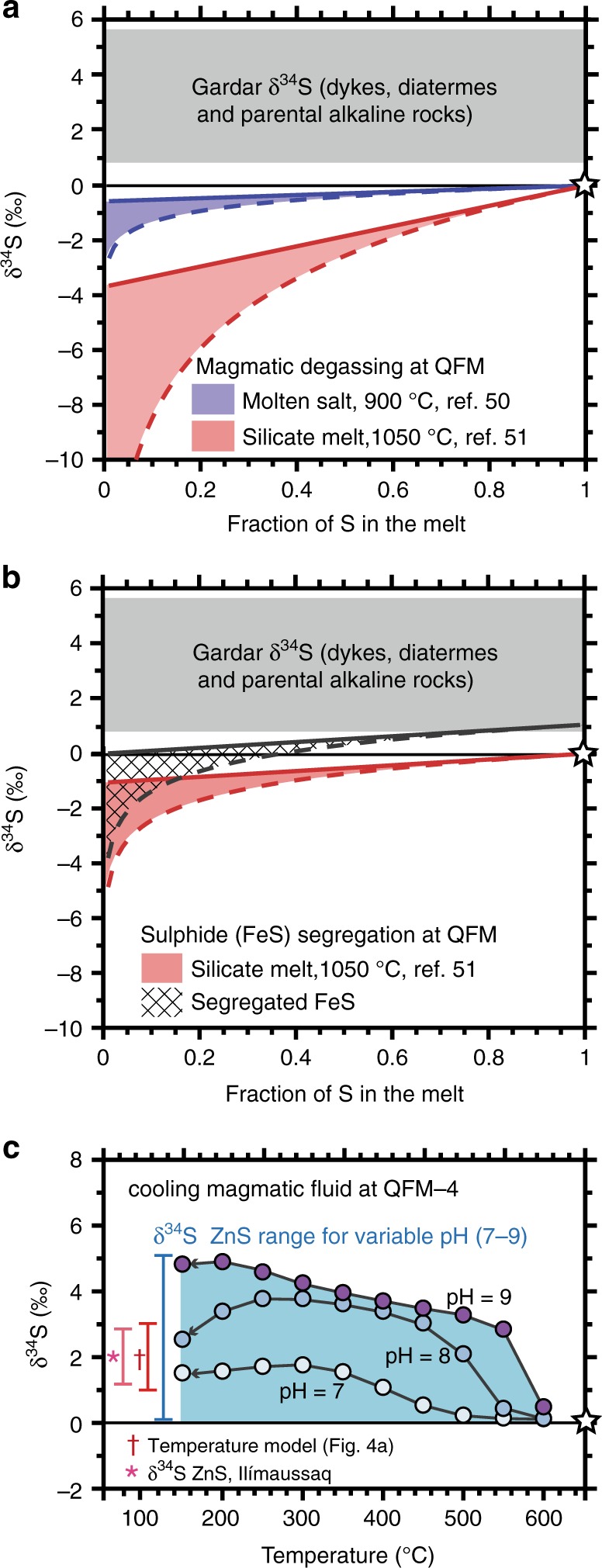
The impacts of magmatic degassing, sulphide segregation and pH variation on sulphur isotopes. All models are relevant to Gardar magma storage conditions (see text for discussion) and grey shaded regions show the δ^34^S range of primitive Gardar magmas. In **a** changing δ^34^S of residual melt due to degassing is shown for magmas at QFM with initial δ^34^S shown by the white star. Degassing-induced fractionation was calculated using the equations of ref. ^35^ and melt S speciation models of ref. ^77^. Dashed and solid lines represent open and closed-system degassing^76^, respectively, with empirical fractionation factors from ref. ^50^ (blue, based on molten salts) and ref. ^51^ (red, from silicate melts). In both cases, degassing imparts negative-δ^34^S fractionation in the residual melt. In **b** the effect of sulphide (FeS) segregation on the δ^34^S of the silicate melt (red) and separated FeS (hatched field) is shown, for open and closed-system models (solid and dashed lines, respectively). We only show the models generated using the fractionation factors of ref. ^51^ (for figure clarity and because ref. ^50^ results were virtually identical). In **c** we model the δ^34^S of sphalerite (ZnS) precipitated by an extremely reduced magmatic fluid (QFM–4) with variable pH (7–9), analogous to agpaitic rocks of Ilímaussaq^43,56^. These calculations follow the equations of ref. ^36^ (see Methods). At high pH the δ^34^S of ZnS is ~5‰ higher than the bulk magmatic fluid (δ^34^S_∑S_ = 0‰, white star) due to the S speciation and dominance of S^2−^. However, Ilímaussaq ZnS δ^34^S shows a limited range (bar labelled with asterisk) and a much better overlap with models that only take account of temperature (bar labelled with dagger), see Fig. 4a and text for full discussion

S isotope fractionation due to magmatic degassing and sulphide segregation is strongly dependent on redox conditions. Fortunately, fO_2_ in our mafic dyke and alkaline intrusion samples are well-constrained^14,40,43,49^ and they are accepted to have formed at conditions at or below QFM. In Fig. 3a we evaluate variations in melt δ^34^S as a function of degassing at QFM using numerical models of ref. ^35^ and fractionation factors of refs. ^50,51^. Calculations suggest that reduced S species S^2–^ and H_2_S dominate the melt and gas, respectively, and predict decreasing melt δ^34^S as a consequence of degassing. The magnitude of δ^34^S change (0–10‰) depends on the extent of degassing, choice of fractionation factors (molten salts^50^ vs. silicate melts^51^) and degassing scenario (open- vs. closed-system degassing^35^). Given that our analyses of primitive dykes, diatremes and early-formed magmatic rocks show that they are S-rich (>500 ppm) with overwhelmingly positive δ^34^S (1–5‰), we conclude that their magmatic source must be >1‰ and infer that degassing has not significantly shifted isotopic values.

Although measurements of δ^34^S in mafic rocks suggest negligible isotopic fractionation between melt and sulphide mineral phases^52^ we decided to evaluate sulphide (FeS) segregation at fO_2_ conditions relevant to the Gardar (using equations of ref. ^35^ and fractionation factors for silicate melts^51^, Fig. 3b). Like magmatic degassing, sulphide segregation decreases δ^34^S in the residual melt (0–5‰) and cannot explain the positive values observed in primitive magmatic rocks and early stages of the alkaline intrusions (Fig. 2a, b, d, f). Moreover, when comparing δ^34^S of magmatic cumulate with late-stage melts for Ilímaussaq and Motzfeldt samples, we find their values are indistinguishable (mostly within 1‰, Supplementary Data 2). Thus, our observations and models indicate that sulphide segregation played a negligible role modifying δ^34^S of Gardar melts.

Having established that crustal assimilation, degassing and sulphide segregation had minimal effects on melt δ^34^S, we now consider how changes in temperature-pH-fO_2_ impact mineral δ^34^S. Given the isotopic and mineralogical distinctions between the early- and late-stage samples (Fig. 2), we consider these groups separately when modelling these processes.

**Fig. 4:**
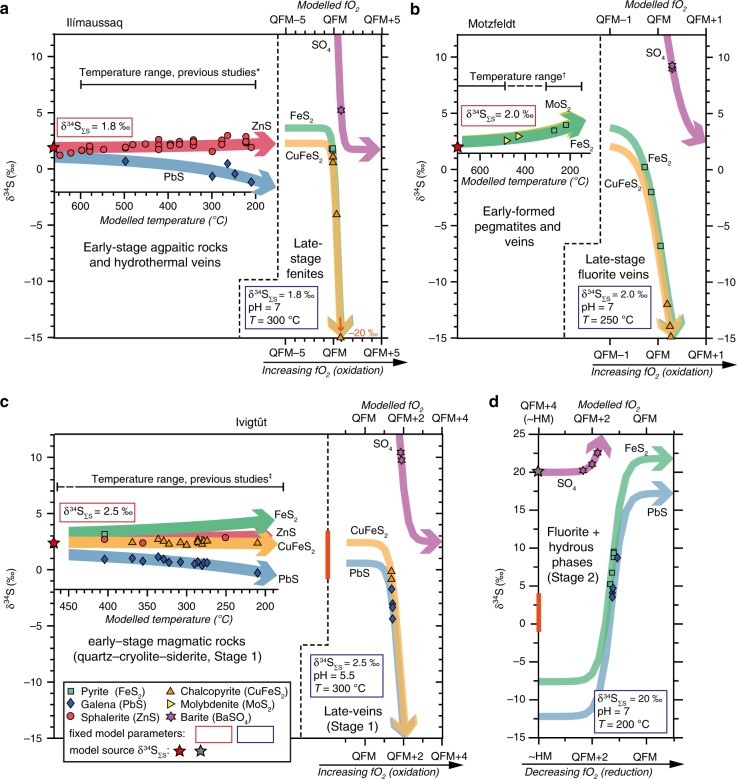
Sulphur isotope results for the Gardar alkaline intrusions. **a** Ilímaussaq, **b** Motzfeldt and **c** and **d** Ivigtût. Data symbols and colours correspond to the sulphide or sulphate mineral analysed. Isotopic fractionation caused by changing temperature and fO_2_ are shown as the bold lines and are specific to each mineral phase. Temperature variations were modelled using compiled fractionation factors^53^, while fO_2_ variations applied the methods of ref. ^36^. Fixed model parameters are shown in white boxes and detailed in the Supplementary Discussion. Note that **d** has a different scale from the other plots and so an orange bar is used to orient the reader to the range of the Ivigtût magmatic fluids in **c**. High-δ^34^S values observed in the final-stage fluorite and hydrous phases of Ivigtût cannot be explained by oxidation of a magmatic fluid (with δ^34^S_∑S_ = 2.5‰, **c**), and so an alternative model involving reduction of an externally derived brine with δ^34^S_∑S_ = 20‰ is proposed (**d**, see text for discussion). The temperature range of previous studies (from phase equilibria, mineral chemistry and fluid inclusions) is shown as the black horizontal bar and is solid where multiple data sets agree and dashed where temperature constraints are sparse. Relevant references for each complex are given by the symbols: asterisk indicates refs. ^46,54–56^; dagger indicates ref. ^40^; and double dagger indicates refs. ^14,42,57^

Early magmatic rocks and veins contain only sulphide (Fig. 2b, d, f) and in a few samples from Ilímaussaq and Ivigtût we identified multiple sulphide phases. This allows us to quantify temperature variations using isotope geothermometry^53^. At Ilímaussaq, sphalerite-galena pairs yield temperatures of 500–200 °C (Fig. 4a), in agreement with temperature estimates from fluid inclusions^54,55^ and phase equilibria^56^ (600–200 °C). At Ivigtût, sulphides from the early-formed magmatic rocks support temperatures of 400–200 °C, also consistent with independent temperature constraints^14,42,57^ (Fig. 4c). To evaluate whether temperature variations explain the observed δ^34^S range in all early-stage samples, we modelled δ^34^S fractionation for different sulphide minerals at each intrusion (shown as the coloured arrows in left hand plots in Fig. 4a–c). At Ilímaussaq and Ivigtût, an elevated δ^34^S_∑S_ of 1.8‰ and 2.5‰, respectively, provides a good fit, encompassing almost all samples with mineral pairs and individual sulphides where temperatures are unconstrained. Although sulphide mineral pairs were absent in early-stage Motzfeldt samples (Fig. 2b) the high-δ^34^S of molybdenite and pyrite (2.6–3.9‰) again suggests a high-δ^34^S source (δ^34^S_∑S _≈ 2‰) since falling temperatures increase sulphide δ^34^S by only ~1.5‰ (Fig. 4b). Thus, early magmatic sulphides at Ilímaussaq, Motzfeldt and Ivigtût require an elevated δ^34^S_∑S_ (1.8–2.5‰) to yield credible estimates on formation temperatures (Fig. 4a–c).

Although variable temperatures account for the δ^34^S range in early-stage sulphides, it is important also to consider pH, which controls S speciation (i.e., the abundance of H_2_S, HS^-^, and S^2−^) and might impart isotopic fractionation in a reduced magmatic fluid^36^ (Supplementary Fig. 2). Ours and previous modelling^36^ (Supplementary Fig. 3) show that when pH is <7, H_2_S is the dominant S fluid phase and there is minimal fractionation between the fluid and the precipitated sulphide. At Ivigtût and Motzfeldt pH was likely <7 (see Supplementary Discussion) and so these effects can be ignored. However, at Ilímaussaq phase equilibria^56^ have been used to argue that the agpaitic magmatic fluids had high and potentially variable pH ≥ 7. In Fig. 3c, we calculate how δ^34^S of ZnS (the main sulphide phase in Ilímaussaq rocks, Fig. 2b) varies during cooling when δ^34^S_∑S_ is fixed (0‰) and pH is variable (7–9). These models predict ZnS δ^34^S of 0–5‰, which is much greater than the δ^34^S range observed in natural samples from Ilímaussaq (pink bar, Fig. 3c). These models also predict that at high temperatures, δ^34^S should converge to the bulk δ^34^S_∑S_ of 0‰, a feature not seen in our samples and contrary to Ilímaussaq whole-rock δ^34^S, which are mostly between 1 and 2‰ (Table 1). We conclude that the close correspondence between ZnS δ^34^S from Ilímaussaq and the temperature modelling results in Fig. 4a (also shown as the red bar in Fig. 3c) support temperature as the main control on δ^34^S. Varying temperature provides the best explanation of all early-stage δ^34^S (Fig. 4) and also requires an elevated source δ^34^S, consistent with all whole-rock δ^34^S observations from the Gardar (1–5‰).

Unlike the early-stage samples, late-stage veins and fenites (Fig. 2c, e, g) contain sulphates and sulphides. The difference between sulphate and sulphide δ^34^S is up to 15–25‰ in these late-stage samples (Fig. 4a–d) and suggests low temperatures of formation 200–300 °C (for reference isotope fractionation at magmatic temperatures, ~600 °C, is ~8‰^50^). Sulphates require increased concentrations of oxidised S species (SO_4_^2–^), suggesting that late magmatic fluids underwent an fO_2_ increase. Models calculating δ^34^S variations from oxidation of a magmatic fluid are shown in Fig. 4a–c (right hand panels). Oxidation causes sulphate to become the dominant S phase and approach the δ^34^S_∑S_ value. At equilibrium, differences in bond stiffness between oxidised and reduced S species favour heavy ^34^S isotope substitutions in sulphate^58^ and lead to a sharp δ^34^S decrease in co-existing sulphides. Increasing fO_2_ adequately explains both the appearance of barite and the negative isotopic shifts in late-stage sulphides (Fig. 4a–c and Supplementary Fig. 4). Late-stage oxidation was most likely driven by an influx of externally derived fluids and at both Ilímaussaq and Motzfeldt, there is δ^18^O and fluid inclusion evidence^40,46^ for external brines and meteoric fluid infiltrating along the margins of the intrusions (where our late-stage minerals were sampled).

At Ivigtût late-stage sulphides reveal both low- and high-δ^34^S. Barites also show groupings of ~10 and ~21‰ (Fig. 2g). While oxidation of a reduced alkaline fluid, by infiltration and mixing with an external brine^14^, explains the low-δ^34^S sulphides in late-stage veins (Fig. 4c and Supplementary Fig. 5) it cannot explain the array of high-δ^34^S sulphides associated with fluorite and hydrous phases (Fig. 4d, note different scale from other panels). To account for these high-δ^34^S samples we envisage a reverse process, whereby an infiltrating brine with Mesoproterozoic seawater-like δ^34^S (δ^34^S_∑S_ = 20‰) is reduced on mixing with the magmatic fluids (Supplementary Fig. 6). As noted above, differences in bond stiffness between oxidised and reduced S species favour heavy isotope substitutions in the sulphate^58^ (Fig. 4d) and so our model rationalises the presence of barite with δ^34^S up to 22.5‰ as well as the pyrite and galena with elevated and wide-ranging δ^34^S (since small variations in redox lead to large isotopic shifts, 3–10‰, Fig. 4d). Although these final-stage sulphates and sulphides are rarely found in direct contact (and do not provide unequivocal evidence for equilibrium), our model complies with previous evidence for late-stage brine influx (e.g., fluid inclusions^14^), and strengthens the case that Ivigtût represents a heterogeneous mixing zone between a reduced CO_3_^2−^ and F^−^ rich magmatic fluid (δ^34^S_∑S_ = 2.5‰) and oxidised brine (δ^34^S_∑S_ = 20‰). Finally, although we invoke external fluids as a cause for late-stage oxidation at Ilímaussaq and Motzfeldt, at Ivigtût a greater ratio of external to internally derived fluids is required, reflecting the much smaller size of Ivigtût (~300 m in diameter), compared to Ilímaussaq and Motzfeldt (km-scale).

### Sulphur isotope signature of the Gardar magma source

Primitive dykes and diatremes provide the best constraints on the Gardar mantle source^41^ and all possess high-δ^34^S (1–5‰). Magmatic cumulate and dykes from the alkaline intrusions (Fig. 2b, d, f) also show positive δ^34^S and, like the primitive dyke and diatreme samples, appear unaffected by crustal contamination, magmatic degassing and sulphide segregation (Table 1 and Fig. 3a, b). This has two important implications. First, because the δ^34^S source values calculated for early-stage sulphide-dominated rocks from the alkaline intrusions (Fig. 4a–c) are within ~1‰ of their parental magmatic units (Fig. 2), and also overlap the δ^34^S of primitive samples; this provides strong evidence that evolved alkaline rocks constrain magma source δ^34^S. This conclusion resonates with earlier studies^30^ and suggests that, because alkaline rocks are exceptionally S-rich, their mineral δ^34^S can be used to evaluate source δ^34^S (i.e., δ^34^S_∑S_). The main caveat is that S minerals must be dominated by either reduced or oxidised phases. Only with our complete data set from multi-phase alkaline intrusions and their associated primitive magmas has it been possible to verify fully this hypothesis.

The second implication is that Gardar magma sources are enriched in δ^34^S. Gardar δ^34^S is well above the accepted range for the asthenospheric upper mantle (−1 ± 0.5‰^59^) and shows closer correspondence to subduction zone settings^1^, implying there is recycled surface S in their source. This is coherent with virtually all prior geochemical investigations of the Gardar, which have advocated a significant involvement of a metasomatised SCLM component in magmagenesis^15,38^ (originating from subduction process ~500 Ma before rift onset^22,39,40^). While it is clear that Gardar mantle metasomatism resulted from subduction processes^38^, our δ^34^S analyses cannot determine the precise source (i.e., slab-derived melts or pore fluids). Trace elements of primitive Gardar melts (e.g., Th/Ce ratios) have been used to argue for fluid-dominated metasomatism^15,38^, while recent δ^34^S analyses of arc cumulates^60^ suggest that sulphate-rich pore fluids are preferentially driven off at the arc front and imply that slab-derived melts are the key metasomatic agent. Although future δ^34^S investigations of Gardar mantle xenoliths may address this issue, the salient point is that alkaline magmas encode information about the fate of surface S subducted into the mantle.

### The origin and evolution of alkaline magmas

**Fig. 5:**
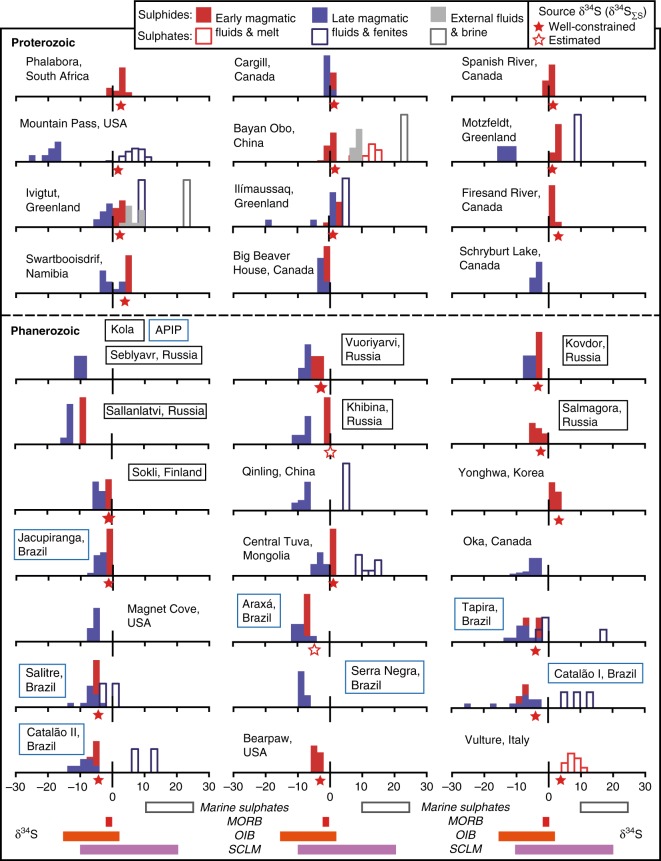
Global compilation of sulphur isotopes from alkaline magmas. Complexes are arranged from oldest (top left) to youngest (bottom right). Normalised histograms show early- (red) and late-stage (blue) δ^34^S from sulphide and sulphate minerals, filled and outlined bars, respectively. Early-stage reflects high temperatures, reduced conditions and general absence of sulphate minerals (vice-versa for late-stage). δ^34^S analyses of fenites, alkaline metasomatic fluids found in the country rocks, are included with the late-stage (blue) histograms. Grey bars correspond to late-stage high-δ^34^S sulphate and sulphide sources that suggest an external (seawater-like) source. Red stars represent the magma source δ^34^S, and are filled where there are abundant samples and petrographic evidence for either a sulphate or sulphide-dominated assemblage, while unfilled stars represent our best-estimate when samples are few and there is sparse information on S mineral abundance. The stars indicate a source uncertainty of ±1.5‰, which is a large but realistic uncertainty (see text for discussion). The δ^34^S of potential S sources are shown by the bars at the base of the plot and include: marine sulphates^9^; mid-ocean ridge basalt (MORB)^59^, ocean island basalt (OIB)^2,3,6^ and sub-continental lithospheric mantle (SCLM)^63^. Data and references are provided for the ~600 samples in Supplementary Data 1–3

Our detailed study of the Gardar intrusions shows that, despite major differences in the origin of the magmatic fluids, S isotopes are fractionated by common evolutionary processes. To explore whether similar redox changes and magma source δ^34^S typify other alkaline systems we compiled a global data set (Fig. 5).

Our compilation reveals that alkaline rocks span an exceptionally wide δ^34^S range (–25 to +25‰, Fig. 5). Almost invariably, there is a decrease in δ^34^S between early and later formed sulphides within a given system. Only at Ivigtût and Bayan Obo were late-stage sulphides with high-δ^34^S (~9‰) and sulphates with seawater-like δ^34^S (>20‰) observed. These data require significant external brine influx and reduction in a magmatic-hydrothermal environment. At Ivigtût we suggest this took place concurrently with the magmatic phase, while at Bayan Obo a more complex multi-stage evolution is envisaged^61^ involving Mesoproterozoic and Palaeozoic hydrothermal events.

Sulphur isotope shifts between early and late-stage alkaline rocks have been reported at individual systems^30–33^ and our global compilation demonstrates the ubiquity of this process. Early stages are almost always reduced and sulphide-dominated (Fig. 5). With cooling, the reduced magmatic systems collapse, oxidised S (sulphate) becomes dominant and this leads to a major δ^34^S decrease in latest sulphide minerals. It is important to note that while external oxidising fluids were implicated in our study, previous authors^30,62^ have suggested that alkaline melts might also show a relative fO_2_ increase (i.e., oxidation) with falling temperatures through natural variations in the crystallising assemblage. Although the precise cause of late-stage oxidation at each complex is beyond the scope of this study, our compilation (Fig. 5) shows that δ^34^S is exceptionally sensitive to low-temperature oxidation and is an excellent tool for fingerprinting these fundamental changes in fluid chemistry at all alkaline systems.

Our δ^34^S observations alongside previous studies^30^ emphasise that S-rich alkaline rocks can be used to evaluate magma source δ^34^S when they are dominated (>90%) by either sulphide or sulphate. While measurements of primitive alkaline magmas are undoubtedly the best method for determining magma source δ^34^S, our detailed case study demonstrates that a wide variety of alkaline rocks, including magmatic cumulate, late-stage silicate melts, carbonatites and aluminofluoride melts (Ivigtût), closely approximate source δ^34^S (within ~1‰). Given that virtually all alkaline systems mirror the δ^34^S trends observed in the Gardar (Fig. 5) it is reasonable to assume that the isotope systematics that govern Gardar melts are applicable elsewhere. Thus, for each alkaline system in Fig. 5 we averaged δ^34^S in the most primitive, high temperature (≫300 °C) phases (mostly magmatic cumulate) to estimate magma source δ^34^S. We exclude sulphide minerals that show large isotopic fractionation at high temperatures (i.e., galena^53^), and where multiple sulphide minerals were reported we include only the most reduced phase (e.g., taking pyrrhotite over pyrite) and apply temperature corrections similar to our Gardar study (Fig. 4a–c). Well-constrained source values (Fig. 5) were only calculated for systems that met these criteria and where petrological observations confirm that reduced or oxidised S dominated the mineralogy. Based on our case study, where >95% of all early-formed S minerals (excluding galena, above) are within ±1.5‰ of the most primitive δ^34^S values, we propose similar large but reasonable uncertainties on our magma source δ^34^S (Fig. 5).

Adopting this approach, we find that the δ^34^S of alkaline magma sources fall between –5 and +5‰ (Fig. 5). As noted previously, mid-ocean ridge basalts (MORB) have δ^34^S of −1 ± 0.5‰^59^, and although we do not exclude mixing of multiple mantle sources, a key observation is that a simple asthenospheric upper mantle source cannot explain the global δ^34^S diversity of alkaline magmas. Alkaline magmas show much closer correspondence to OIB^2,3,6^ and SCLM^63^ (Fig. 5), and require mantle reservoirs with both enriched and depleted δ^34^S. Recycled surface S is the most logical candidate and is consistent with findings from trace elements, radiogenic (Sr–Nd–Pb) and stable isotopes (B), which often require a recycled crustal component in alkaline magma sources^13,16,17,24^. Although alkaline rocks originate through a variety of mantle processes, including mantle plumes^19^ and subduction-related mantle metasomatism^38^, they undoubtedly play a key role in returning previously subducted S to the surface, and are therefore an integral component of the global S cycle.

Our compilation also shows strong evidence for regional variations in source δ^34^S. Gardar intrusions have source δ^34^S of 1–3‰, similar to Proterozoic carbonatites from Canada^64^ (Fig. 3), but starkly contrasting with the negative-δ^34^S suggested from Russia and Finland (Kola Alkaline Province^33,65,66^) and Brazil (Alto Paranaiba Igneous Province, APIP^32^). We infer that these data represent genuine low-δ^34^S sources because: (1) δ^34^S is consistent between multiple complexes at a regional scale; (2) different regional studies provide consistent isotope values (e.g., all studies of Kola sulphides^33,65,66^ show isotopically light values); and (3) individual mineral δ^34^S are exceptionally low (e.g., sulphates are always enriched in ^34^S and should have δ^34^S ≥ 0‰, however, barites from Salitre and Tapira in APIP^32^ possess negative-δ^34^S, requiring a δ^34^S_∑S_ ≪ 0‰). Additionally, at Kola and APIP, isotopic studies^21,33,67,68^ have ruled out assimilation of local Precambrian crust. The regional variations in source δ^34^S (Fig. 5) imply that the processes that enrich the mantle prior to alkaline magmatism, whether SCLM metasomatism or plume-related, are isotopically diverse and take place at a regional scale.

### Links to the global sulphur cycle

**Fig. 6:**
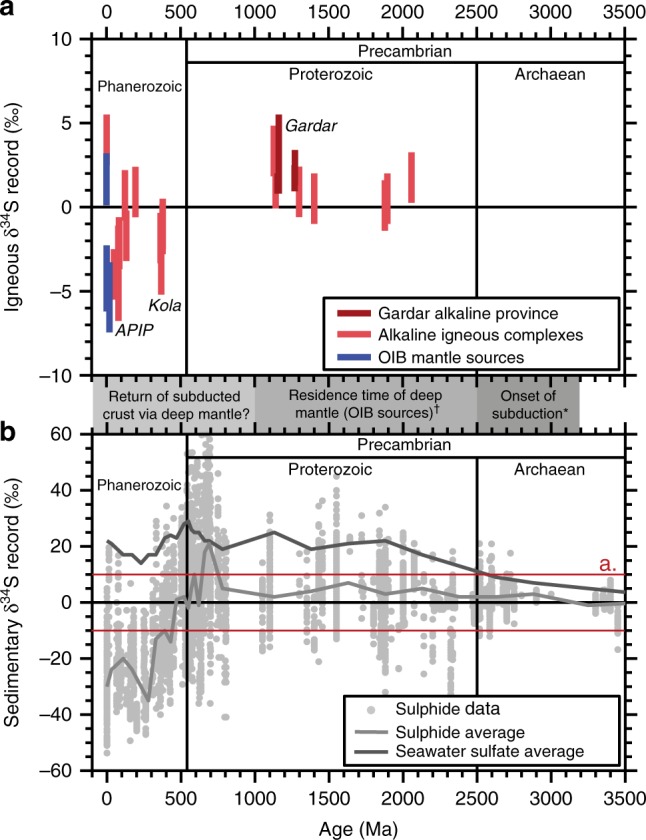
Comparison of igneous and sedimentary δ^34^S records. In **a** red bars represent the magma source δ^34^S compilation from this study (Fig. 5), with an uncertainty of ±1.5‰. Only complexes with well-constrained source δ^34^S are shown (see text for details). Blue bars are δ^34^S values of the Mangaia^2^, Pitcairn^3^, Discovery^5^, Samoa^6^ and Canary Islands^7^ ocean island basalts (OIBs). OIBs show geochemical signatures of recycled crust and have both positive and negative-δ^34^S, akin to alkaline rocks. Notable low-δ^34^S alkaline provinces, associated with Phanerozoic mantle plumes are also labelled, and include Kola^19^ and the Alto Paranaiba Igneous Province^21^ (APIP). In **b** sulphide δ^34^S records for sedimentary rocks from continental shelves and inland seas, as well as seawater sulphate δ^34^S are from the compilation of ref. ^10^. The red inset corresponds to the scale in **a**. Mantle geodynamic timescales are also included in the time-series and suggest development of modern-style subduction at 3.2–2.5 Ga (asterisk indicates refs. ^71–74^), deep mantle (beneath 400 km) residence times of ~1.5 Ga (dagger indicates ref. ^75^), and would predict return of deep subducted crust after ~1 Ga

The alkaline rocks compiled here span an age range of ~2060 to ~0 Ma and provide a δ^34^S time-series of magma sources (Fig. 6). While OIBs are limited by the age of oceanic crust (<200 Ma), alkaline magmas that intruded continental crust are well-preserved over much of Earth history and provide a valuable inventory of mantle evolution^17,24^. Our igneous δ^34^S time-series shows a temporal trend with Proterozoic alkaline magmas largely restricted to positive δ^34^S values (0–5‰) and Phanerozoic suites showing greater δ^34^S diversity (–5 to 4‰, overlapping the range of OIB sources).

Our igneous δ^34^S compilation also shows a first-order correspondence with the global sedimentary sulphide δ^34^S record from continental shelves and inland seas (Fig. 6). Pyrite from sedimentary rocks shows a long-term δ^34^S evolution from zero to slightly positive values in the Archaean and Proterozoic to significantly lower δ^34^S in the Phanerozoic. This record of oceanic δ^34^S represents a shift from an anoxic deep ocean with a small sulphate reservoir to more oxygenated deep waters with a large sulphate reservoir after ~600 Ma, coinciding with the onset of bioturbation^10^. Although Proterozoic sedimentary δ^34^S is on average positive, pyrite with negative-δ^34^S is mainly observed in deeper parts of sedimentary basins^69^. These observations have led several workers to invoke a missing Proterozoic ^34^S-depleted S pool, deposited in deep water settings and lost from the surface via subduction^9,70^.

**Fig. 7:**
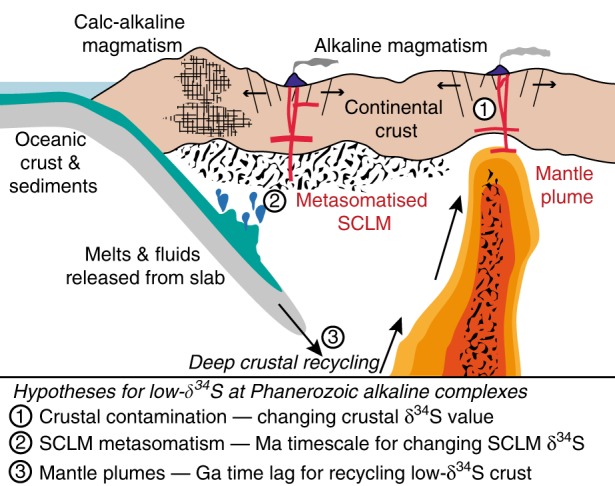
Schematic summarising the new constraints on alkaline magma sources. Hypotheses to explain the occurrence of low-δ^34^S Phanerozoic alkaline complexes (Kola and APIP) and the secular change in magma δ^34^S are discussed in the text

Our observations raise two key questions: why do igneous and sedimentary δ^34^S show similar time-evolving trends, and why are anomalous low-δ^34^S alkaline provinces only observed in the Phanerozoic? In Fig. 7 various scenarios are summarised. In the first case, the igneous δ^34^S pattern could simply reflect crustal assimilation of sedimentary rocks, i.e., because Phanerozoic sedimentary crust has low-δ^34^S (Fig. 6), assimilation would lead to low-δ^34^S values in some Phanerozoic magmas. The second hypothesis assumes alkaline magmas are derived from metasomatised SCLM, and that secular variation in δ^34^S reflects changing composition of the SCLM. Since subduction exerts an important control on SCLM composition, this would suggest that low-δ^34^S sedimentary rocks were subducted in the Phanerozoic, imprinted a low-δ^34^S signature on the SCLM, and were tapped by Phanerozoic magmas within ~10–100 Ma. A final hypothesis is that during the Precambrian (both Proterozoic and Archaean) low-δ^34^S crust was subducted and stored in the deep mantle^8,9,70^, hence the secular evolution represents a Ga time-lag in returning this material to the surface.

Focusing on the anomalous low-δ^34^S alkaline provinces, i.e., Kola and APIP, it is easy to eliminate crustal assimilation because the local crust is Precambrian and isotopic studies reject crustal interactions^21,33,67,68^. Discriminating between the other scenarios is more challenging, particularly because δ^34^S alone does not allow unambiguous discrimination between deep mantle (OIB) and SCLM sources (Fig. 5). Nevertheless, if both Kola and APIP sources were modified by low-δ^34^S sediments subducted in the Phanerozoic, then sutures (former subduction zones) of Phanerozoic age should be present. In Kola and APIP the nearest sutures are Palaeo- and Neoproterozoic, respectively, ruling out Phanerozoic subduction-related source modification.

We suggest that low-δ^34^S Phanerozoic alkaline magmas at Kola and APIP are derived from a mantle plume with a low-δ^34^S signature. Importantly, noble gas isotope signatures support a deep plume source at both Kola^19^ and APIP^18^, and radiogenic isotopes also support ancient (potentially Archaean) sources at Kola^33^. Thus, our favoured hypothesis is that their low-δ^34^S signatures reflect deep recycling of previously subducted low-δ^34^S crust of either Archaean or Proterozoic age. This implies that the co-variation of igneous and sedimentary δ^34^S (Fig. 6) is fortuitous since low-δ^34^S provinces reflect recycling ancient (Ga) rather than contemporary (Ma) S. Our suggestion that low-δ^34^S alkaline provinces are a Phanerozoic phenomenon is also consistent with timescales of Earth’s geodynamic cycle, i.e., plate tectonics and subduction into the deep mantle initiated around 3.2–2.5 Ga^71–74^, residence timescales in the lower mantle are calculated to be on the order of 1.5 Ga^75^, thus we would expect to see deep crustal recycling after ~1 Ga (Fig. 6).

Although our final hypothesis complements several OIB studies^2,3^, which have confirmed recycled Archaean S with characteristic negative-δ^34^S, other OIB studies have reported positive-δ^34^S (Fig. 6) and linked these to recycled Proterozoic S^5–7^. While we do not suggest that all recycled Proterozoic and Archaean crust has negative-δ^34^S, our observations require a deep mantle source and, as has been advocated at other OIB sources^2,3,8^, this is most plausibly linked to low-δ^34^S recycled crust. Likewise, we do not rule out plume origins for the Proterozoic alkaline provinces^20^, but emphasise that they do not carry a low-δ^34^S signature^2,3,8,9^.

Ultimately, our global compilation represents an important new δ^34^S time-series of mantle source evolution. Key to unravelling the igneous record is determining whether specific δ^34^S signatures reflect a deep mantle plume or metasomatised SCLM origin. Further studies should look to combine multiple S isotopes, stable isotopes, radiogenic isotopes and noble gases for the same sample suite. This approach will clarify mantle sources and the temporal patterns in Fig. 6, and provide robust geochemical constraints on the connectivity of Earth’s surface and mantle S reservoirs.

## Methods

### Sulphur isotopes

We extracted visible S-bearing minerals using a microdrill. For samples without visible S-bearing minerals we generated a whole-rock powder and extracted sulphide phases as Ag_2_S using a Cr reduction procedure^47^. S isotope analysis (δ^34^S) was undertaken at three institutes: Scottish Universities Environmental Research Centre (SUERC, East Kilbride, UK); School of Earth and Environmental Sciences (University of St Andrews, UK) and Fachbereich Geowissenschaften (Universität Tübingen, Germany). An Isoprime VisION isotope ratio mass spectrometer (IRMS) with a linked Vario PYRO cube elemental analyser (EA) was used at SUERC, a Thermo Quest Delta + XL IRMS connected to a NC 2500 was used at Tübingen, and at St Andrews we used an EA IsoLink, coupled to a MAT 253 IRMS via a Conflo IV. Standards were closely spaced throughout the runs and used to calibrate sample isotope compositions. Replicates between the different institutes were consistent to the error within natural δ^34^S heterogeneity (generally, ±0.3‰ at 2 s.d. for the early-formed high temperature, >300 °C, sulphides).

### Sulphur concentrations

Sulphur concentrations in whole-rock powders were determined via Combustion Ion Chromatography (CIC) at the Universität Tübingen (Germany) using a 930 Compact IC Flex chromatograph (Metrohm) combined with a combustion oven (Analytik Jena). Mixtures of equal amounts (10 mg) of powdered sample and WO_3_ were heated in the combustion oven to 1050 °C in an Ar–O_2_ atmosphere. The loaded steam was condensed and injected into the ion chromatograph equipped with a Metrosep A Supp 5–250/4.0 column (kept at 55 °C) using an NaOH-Na_2_CO_3_-acetone eluent. For the whole analytical procedure, Millipore water (18.2 MΩ.cm) was used and quantification was done using MagIC Net software (Metrohm). The effective detection limit for powdered samples was about 1–2 μg/g and based on the repeated analyses of samples and reference material GSN, relative uncertainties were generally <15% (1 s.d.), depending on the concentrations.

### Isotope modelling

Models of S isotope fractionation due to magmatic degassing and sulphide (FeS) segregation were calculated using the equations of ref. ^35^. In both cases melt δ^34^S is dictated by: total S isotope value (δ^34^S_∑S_); fO_2_ conditions; the fraction of S remaining in the melt; the choice of open vs. closed-system behaviour^76^ and the empirical fractionation factors used. We model all scenarios at QFM since Gardar magmatic suites are accepted to have formed at fO_2_ conditions at or below the QFM buffer^14,40,43,49^. To evaluate S speciation in the melt and gas we used the models of ref. ^77^ and ref. ^35^, respectively. At QFM reduced S species dominate the melt (S^2–^) and gas (H_2_S). Empirical fractionation factors from ref. ^50^ (blue, Fig. 3a) are from experiments on molten salts while those from ref. ^51^ (red, Fig. 3a, b) are from more recent experiments on silicate melts. We expect that the fractionation factors of ref. ^51^ (red, Fig. 3a, b) to be most appropriate for the silicate melts in the Gardar, but have included those of ref. ^50^ because they have traditionally been used^1,35^ and may be suitable for ionic liquids (i.e., carbonatites).

Sulphur isotope fractionation in an evolving magmatic fluid^36^ is primarily controlled by temperature, fO_2_, pH and total S isotope value (or source δ^34^S, δ^34^S_∑S_). These methods are described in detail in by ref. ^36^ and were used here to evaluate how changes in temperature-fO_2_-pH may have generated the isotope fractionation in our samples (Figs. 3c and 4). It is important to note that although we used identical equations of ref. ^36^, we updated the fractionation factors using a more recent compilation^53^, as well as activity coefficients and equilibrium constants from the SUPCRTBL^78^ and LLNL data sets (note that the latter is included in *The Geochemists Workbench* software^79^).

To understand the geological feasibility of the predicted isotope fractionation it is essential to also evaluate mineral stability fields^36^. We used *The Geochemists Workbench*^79^ to calculate pH–fO_2_ phase diagrams for simplified element systems, e.g., Fe–Cu–S–O, Pb–S–O and Zn–S–O, and overlay these on the modelled isotope variations (Supplementary Discussion). Previous fluid inclusion, mineralogical and phase equilibria studies of Ilímaussaq, Motzfeldt and Ivigtût intrusions are available (detailed above and in the Supplementary Discussion) and provide valuable constraints on the temperature, pH and fO_2_ conditions. In the Supplementary Discussion we outline these constraints for each system and justify our preferred trajectories that explain both the isotopic and mineralogical changes observed in Fig. 4.

## Supplementary information

Supplementary Information

Peer Review File

Description of Additional Supplementary Files

Supplementary Data 1

Supplementary Data 2

Supplementary Data 3

## Data Availability

The data that support the findings of this study are available within the article and its Supplementary Information files.

## References

[1] De Hoog JCM, Taylor BE, Van Bergen MJ (2001). Sulfur isotope systematics of basaltic lavas from Indonesia: implications for the sulfur cycle in subduction zones. Earth Planet. Sci. Lett..

[2] Cabral RA (2013). Anomalous sulphur isotopes in plume lavas reveal deep mantle storage of Archaean crust. Nature.

[3] Delavault H, Chauvel C, Thomassot E, Devey CW, Dazas B (2016). Sulfur and lead isotopic evidence of relic Archean sediments in the Pitcairn mantle plume. Proc. Natl Acad. Sci..

[4] Farquhar J, Wing BA (2003). Multiple sulfur isotopes and the evolution of the atmosphere. Earth Planet. Sci. Lett..

[5] Labidi J, Cartigny P, Moreira M (2013). Non-chondritic sulphur isotope composition of the terrestrial mantle. Nature.

[6] Labidi J, Cartigny P, Jackson MG (2015). Multiple sulfur isotope composition of oxidized Samoan melts and the implications of a sulfur isotope ‘mantle array’ in chemical geodynamics. Earth Planet. Sci. Lett..

[7] Beaudry P (2018). Degassing-induced fractionation of multiple sulphur isotopes unveils post-Archaean recycled oceanic crust signal in hotspot lava. Nat. Commun..

[8] Farquhar J, Jackson M (2016). Missing Archean sulfur returned from the mantle. Proc. Natl. Acad. Sci..

[9] Farquhar J, Nanping WU, Canfield DE, Oduro H (2010). Connections between sulfur cycle evolution, sulfur isotopes, sediments and base metal sulfide deposits. Econ. Geol..

[10] Canfield DE, Farquhar J (2009). Animal evolution, bioturbation, and the sulfate concentration of the oceans. Proc. Natl Acad. Sci..

[11] Jones AP, Genge M, Carmody L (2013). Carbonate melts and carbonatites. Rev. Mineral. Geochem..

[12] Bell K, Simonetti A (2010). Source of parental melts to carbonatites–critical isotopic constraints. Mineral. Petrol..

[13] Hoernle K, Tilton G, Le Bas MJ, Duggen S, Garbe-Schönberg D (2002). Geochemistry of oceanic carbonatites compared with continental carbonatites: mantle recycling of oceanic crustal carbonate. Contrib. Mineral. Petrol..

[14] Köhler J, Konnerup-Madsen J, Markl G (2008). Fluid geochemistry in the Ivigtut cryolite deposit, South Greenland. Lithos.

[15] Bartels A, Nielsen TF, Lee SR, Upton BGJ (2015). Petrological and geochemical characteristics of Mesoproterozoic dyke swarms in the Gardar Province, South Greenland: Evidence for a major sub-continental lithospheric mantle component in the generation of the magmas. Mineral. Mag..

[16] Song W (2016). Origin of unusual HREE-Mo-rich carbonatites in the Qinling orogen, China. Sci. Rep..

[17] Hulett SRW, Simonetti A, Rasbury ET, Hemming NG (2016). Recycling of subducted crustal components into carbonatite melts revealed by boron isotopes. Nat. Geosci..

[18] Sasada T, Hiyagon H, Bell K, Ebihara M (1997). Mantle-derived noble gases in carbonatites. Geochim. Cosmochim. Acta.

[19] Marty B (1998). Plume-derived rare gases in 380 Ma carbonatites from the Kola region (Russia) and the argon isotopic composition in the deep mantle. Earth Planet. Sci. Lett..

[20] Ernst RE, Bell K (2010). Large igneous provinces (LIPs) and carbonatites. Mineral. Petrol..

[21] Gibson SA, Thompson RN, Leonardos OH, Dickin AP, Mitchell JG (1995). The late cretaceous impact of the trindade mantle plume: evidence from large-volume, mafic, potassic magmatism in SE Brazil. J. Petrol..

[22] Goodenough KM, Upton BGJ, Ellam RM (2002). Long-term memory of subduction processes in the lithospheric mantle: evidence from the geochemistry of basic dykes in the Gardar Province of South Greenland. J. Geol. Soc. Lond..

[23] Downes H, Balaganskaya E, Beard A, Liferovich R, Demaiffe D (2005). Petrogenetic processes in the ultramafic, alkaline and carbonatitic magmatism in the Kola Alkaline Province: a review. Lithos.

[24] Bell Keith, Tilton George R. (2002). Probing the mantle: The story from carbonatites. Eos, Transactions American Geophysical Union.

[25] Babiel R, Marks MAW, Neumann U, Markl G (2018). Sulfides in alkaline and peralkaline rocks: textural appearance and compositional variations. Neues Jahrb. f.ür. Mineral.-Abhandlungen J. Mineral. Geochem..

[26] Helz GR, Wyllie PJ (1979). Liquidus relationships in the system CaCO3Ca(OH)2CaS and the solubility of sulfur in carbonatite magmas. Geochim. Cosmochim. Acta.

[27] Scaillet B, MacDonald R (2006). Experimental and thermodynamic constraints on the sulphur yield of peralkaline and metaluminous silicic flood eruptions. J. Petrol..

[28] Gold DP (1963). Average chemical composition of carbonatites. Econ. Geol..

[29] Sasaki A, Ishihara S (1979). Sulfur Isotopic Composition of the Magnetite-Series and Ilmenite-Series Granitoids in Japan. Contrib. Mineral. Petrol..

[30] Mitchell RH, Krouse HR (1975). Sulphur isotope geochemistry of carbonatites. Geochim. Cosmochim. Acta.

[31] Drüppel K, Wagner T, Boyce AJ (2006). Evolution of sulfide mineralization in ferrocarbonatite, swartbooisdrif, northwestern Namibia: constraints from mineral compositions and sulfur isotopes. Can. Mineral..

[32] Gomide CS (2013). Sulfur isotopes from Brazilian alkaline carbonatite complexes. Chem. Geol..

[33] Bell K, Zaitsev AN, Spratt J, Fröjdö S, Rukhlov AS (2015). Elemental, lead and sulfur isotopic compositions of galena from Kola carbonatites, Russia–implications for melt and mantle evolution. Mineral. Mag..

[34] Bekker A (2009). Atmospheric sulfur in archean komatiite-hosted nickel deposits. Science.

[35] Marini L, Moretti R, Accornero M (2011). Sulfur isotopes in magmatic-hydrothermal systems, melts, and magmas. Rev. Mineral. Geochem..

[36] Ohmoto H (1972). Systematics of sulfur and carbon isotopes in hydrothermal ore deposits. Econ. Geol..

[37] Upton, B. G. J. Tectono-magmatic evolution of the younger Gardar southern rift, South Greenland. *Geol. Surv. Den. Greenl. Bull*. Vol. 29, 1–124 (Copenhagen, Denmark, 2013).

[38] Köhler J, Schönenberger J, Upton B, Markl G (2009). Halogen and trace-element chemistry in the Gardar Province, South Greenland: subduction-related mantle metasomatism and fluid exsolution from alkalic melts. Lithos.

[39] Marks MAW, Vennemann T, Siebel W, Markl G (2004). Nd-, O-, and H-isotopic evidence for complex, closed-system fluid evolution of the peralkaline Ilı ´ maussaq intrusion, South Greenland. Geochim. Cosmochim. Acta.

[40] Schönenberger J, Markl G (2008). The magmatic and fluid evolution of the motzfeldt intrusion in South Greenland: insights into the formation of agpaitic and miaskitic rocks. J. Petrol..

[41] Coulson IM, Goodenough KM, Pearce NJG, Leng MJ (2003). Carbonatites and lamprophyres of the Gardar Province–a window to the sub-Gardar mantle?. Mineral. Mag..

[42] Pauly, H. & Bailey, J. C. Genesis and evolution of the Igvitut cryolite deposit, SW Greenland. *Meddelser om Grønland Geosci*. **37**, 1–60 (1999).

[43] Marks, M. A. W. & Markl, G. The Ilímaussaq Alkaline Complex, South Greenland. In: Charlier B., Namur O., Latypov R., Tegner C. (eds). *Layered Intrusions*. 649–691 (Springer Geology, Springer, Dordrecht, 2015).

[44] Finch AA (2019). From mantle to motzfeldt: a genetic model for syenite-hosted Ta,Nb-mineralisation. Ore Geol. Rev..

[45] Goodenough KM, Upton BGJ, Ellam RM (2000). Geochemical evolution of the Ivigtut granite, South Greenland: a fluorine-rich ‘A-type’ intrusion. Lithos.

[46] Graser G, Markl G (2008). Ca-rich ilvaite-epidote-hydrogarnet endoskarns: a record of late-magmatic fluid influx into the persodic Ilímaussaq complex, South Greenland. J. Petrol..

[47] Canfield DE, Raiswell R, Westrich JT, Reaves CM, Berner RA (1986). The use of chromium reduction in the analysis of reduced inorganic sulfur in sediments and shales. Chem. Geol..

[48] Hutchison W (2018). The evolution of magma during continental rifting: New constraints from the isotopic and trace element signatures of silicic magmas from Ethiopian volcanoes. Earth Planet. Sci. Lett..

[49] Upton BGJ, Thomas JE (1980). The Tugtutoq Younger Giant Dyke Complex, South Greenland: fractional crystallization of transitional olivine basalt magma. J. Petrol..

[50] Miyoshi T, Sakai H, Chiba H (1984). Experimental study of sulfur isotope fractionation factors between sulfate and sulfide in high temperature melts. Geochem. J..

[51] Fiege A (2015). Experimental investigation of the S and S-isotope distribution between H2O–S ± Cl fluids and basaltic melts during decompression. Chem. Geol..

[52] Labidi J, Cartigny P (2016). Negligible sulfur isotope fractionation during partial melting: evidence from Garrett transform fault basalts, implications for the late-veneer and the hadean matte. Earth Planet. Sci. Lett..

[53] Seal RR (2006). Sulfur isotope geochemistry of sulfide minerals. Rev. Mineral. Geochem..

[54] Konnerup-Madsen J, Rose-Hansen J (1982). Volatiles associated with alkaline igneous rift activity: fluid inclusions in the Ilímaussaq intrusion and the Gardar granitic complexes (south Greenland). Chem. Geol..

[55] Graser G, Potter J, Köhler J, Markl G (2008). Isotope, major, minor and trace element geochemistry of late-magmatic fluids in the peralkaline Ilímaussaq intrusion, South Greenland. Lithos.

[56] Markl G, Baumgartner L (2002). pH changes in peralkaline late-magmatic fluids. Contrib. Mineral. Petrol..

[57] Pauly H (1960). Paragenetic relations in the main cryolite ore of Ivigtut, South Greenland. Neues Jahrb. f.ür. Mineral..

[58] Schauble EA (2008). Applying stable isotope fractionation theory to new systems. Rev. Mineral. Geochem..

[59] Labidi J, Cartigny P, Birck JL, Assayag N, Bourrand JJ (2012). Determination of multiple sulfur isotopes in glasses: a reappraisal of the MORB δ34S. Chem. Geol..

[60] Lee C-TA (2018). Sulfur isotopic compositions of deep arc cumulates. Earth Planet. Sci. Lett..

[61] Liu S (2018). Mesoproterozoic and Paleozoic hydrothermal metasomatism in the giant Bayan Obo REE-Nb-Fe deposit: Constrains from trace elements and Sr-Nd isotope of fluorite and preliminary thermodynamic calculation. Precambrian Res..

[62] Markl G, Marks MAW, Schwinn G, Sommer H (2001). Phase equilibrium constraints on intensive crystallization parameters of the ilimaussaq complex, South Greenland. J. Petrol..

[63] Giuliani A (2016). Sulfur isotope composition of metasomatised mantle xenoliths from the Bultfontein kimberlite (Kimberley, South Africa): contribution from subducted sediments and the effect of sulfide alteration on S isotope systematics. Earth Planet. Sci. Lett..

[64] Farrell S, Bell K, Clark I (2010). Sulphur isotopes in carbonatites and associated silicate rocks from the Superior Province, Canada. Mineral. Petrol..

[65] Grinenko LN, Kononova VA, Grinenko VA (1970). Isotopic composition of sulfide sulfur in carbonatites. Geochemical Int..

[66] Mäkelä M, Vartiainen H (1978). A study of sulfur isotopes in the Sokli multi-stage carbonatite (Finland). Chem. Geol..

[67] Huang Y-M, Hawkesworth CJ, van Calsteren P, McDermott F (1995). Geochemical characteristics and origin of the Jacupiranga carbonatites, Brazil. Chem. Geol..

[68] Lee MJ (2006). Sr-Nd-Pb isotopic compositions of the Kovdor phoscorite-carbonatite complex, Kola Peninsula, NW Russia. Lithos.

[69] Shen Y, Knoll AH, Walter MR (2003). Evidence for low sulphate and anoxia in a mid-Proterozoic marine basin. Nature.

[70] Canfield DE (2004). The evolution of the earth surface sulfur reservoir. Am. J. Sci..

[71] Abbott D, Drury R, Smith WHF (1994). Flat to steep transition in subduction style. Geology.

[72] Shirey SB, Richardson SH (2011). Start of the wilson cycle at 3 Ga shown by diamonds from subcontinental mantle. Science.

[73] Gerya T (2014). Precambrian geodynamics: concepts and models. Gondwana Res..

[74] Laurent O, Martin H, Moyen JF, Doucelance R (2014). The diversity and evolution of late-Archean granitoids: evidence for the onset of ‘modern-style’ plate tectonics between 3.0 and 2.5 Ga. Lithos.

[75] Allègre CJ (2002). The evolution of mantle mixing. Philos. Trans. R. Soc. A Math. Phys. Eng. Sci..

[76] Marini L, Paiotti A, Principe C, Ferrara G, Cioni R (1994). Isotopic ratio and concentration of sulfur in the undersaturated alkaline magmas of Vulture Volcano (Italy). Bull. Volcanol..

[77] Jugo PJ, Wilke M, Botcharnikov RE (2010). Sulfur K-edge XANES analysis of natural and synthetic basaltic glasses: implications for S speciation and S content as function of oxygen fugacity. Geochim. Cosmochim. Acta.

[78] Zimmer K (2016). SUPCRTBL: a revised and extended thermodynamic dataset and software package of SUPCRT92. Comput. Geosci..

[79] Bethke, C. *Geochemical and Biogeochemical Reaction Modeling*. (Cambridge University Press, New York, 2008).

