# Supplementary material for: Sulphur isotopes of alkaline magmas unlock long-term records of crustal recycling on Earth
Source: Nat Commun. 2019 Sep 16;10:4208. doi: 10.1038/s41467-019-12218-1 (PMC6746797; doi:10.1038/s41467-019-12218-1)
Supplement: Supplementary file 1 — Supplementary Information [file 41467_2019_12218_MOESM1_ESM.pdf]

Supplementary Information for

Sulphur isotopes of alkaline magmas unlock long-term records of crustal recycling on Earth

Hutchison et al.

Supplementary Discussion

Modelling S isotope fractionation at the Gardar alkaline intrusions

In the following section we present a detailed summary of the transition between early- and late-stage magmatic fluids at Ilímaussaq, Motzfeldt and Ivigtût (Supplementary Figure 1) based on previous fluid inclusion, mineralogical and phase equilibria studies. These observations are used to constrain fluid evolution trajectories, in terms of temperature-pH-fO_2 and model their isotopic variations (as shown in Figure 4).

Ilímaussaq

The thermo-chemical parameters of magmatic-hydrothermal veins, that host most of the sulphide minerals analysed here, have been the subject of a large number of studies (summarized by ref. [1]). Although the veins display a wide array of textures and mineralogical compositions, quantitative constraints on their formation have been established. For example, the veins were emplaced at 3–4 km (~1 kbar[2]) and fluid inclusions[3] and phase equilibria[4] suggest temperatures of 600–200 °C. The fluids are strongly alkaline with pH >7, and potentially up to 10–12[4,5]. Their fO_2 has been constrained via phase equilibria[5,6] and are consistent with reduced conditions of ΔQFM 0 to –8. These fluids are also characterized by low activities of silica (0.2–0.4) and water (0.5–1)[6], and in our modelling we adopted the S activities used by previous studies[5] (log aS_2 of –2) to maintain consistency with this earlier work.

Phase diagrams were constructed using the above constraints for the simplified systems Fe-S-O, Cu-Fe-S-O, Pb-S-O and Zn-S-O and are shown in Supplementary Figure 2. S isotope ($\delta^{34}S$) fractionation was also calculated (see Methods) and is shown by the coloured background assuming a fixed temperature (300 °C) and $\delta^{34}S_{\sum S}$ (1.8 ‰). The pH and fO_2 constraints on Ilímaussaq's magmatic-hydrothermal veins were used to constrain an initial fluid composition (shown by the asterisk in Supplementary Figure 2) and we evaluated changes in the pH and fO_2 (shown as lines in Supplementary Figure 2, and as model trajectories in Supplementary Figure 3 and Figure 4). The initial composition correctly predicts the stability and isotopic composition of galena and sphalerite in the magmatic-hydrothermal veins, and also indicate the presence of pyrrhotite (which is abundant in Ilímaussaq samples[7,8], but too fine grained to separate for $\delta^{34}S$ analysis).

At Ilímaussaq, oxidized conditions (slightly above the hematite-magnetite, HM, buffer) and temperatures around 200–500 °C were estimated for skarn-like assemblages on the margins of the complex[9]. The metasomatised country rock at the margins of the complex (fenites, Figure 4a) where we sampled pyrite, chalcopyrite and barite also contain abundant epidote and hematite. These samples are very similar to those described by ref. [9], and so we assume similar temperature and fO_2 conditions. These authors[9] attribute the oxidation to an influence of external fluids (likely seawater) entering along the margins of the complex.

It is reasonable to assume that the formation of the reduced magmatic-hydrothermal veins and sulphide-bearing fenites both took place at 300 °C, and so a single phase diagram can be used to understand how changes in pH and fO_2 affect S isotope fractionation (Supplementary Figure 2). Modelled $\delta^{34}S$ variations, corresponding to the arrow vectors in Supplementary Figure 2, are shown below in Supplementary Figure 3. The solid coloured lines indicate oxidation (increasing fO_2) which causes sulphate to become the dominant S phase and approach the $\delta^{34}S_{\Sigma S}$ value. Oxidation leads to a sharp decrease in $\delta^{34}S$ for co-existing sulphides because sulphates favour the heavier ^{34}S isotope. The grey dashed line in Supplementary Figure 3 shows variations in ZnS $\delta^{34}S$ for pH changes between 6 and 10 (at fixed fO_2, Supplementary Figure 2). At or below pH 7, H_2S is the dominant S phase and there is minimal fractionation between the fluid and the precipitated sulphide. Above pH 8 S^{2-} is the dominant S phase and isotopic fractionation between the fluid and the precipitated sulphide is up to 5 ‰, Supplementary Figure 3). This summary of $\delta^{34}S$ variations due to changing fO_2 and pH is relevant to all alkaline intrusions, and for brevity is not repeated for the other complexes.

At Ilímaussaq, changes in pH for a reduced initial fluid (* in Supplementary Figure 2c) cannot explain the appearance of pyrite and barite. Instead, we favour oxidation (increasing fO_2) of the initially reduced magmatic fluids. This explains both the appearance of pyrite and chalcopyrite and their low-$\delta^{34}S$. Note that a green triangle is used in Supplementary Figure 2b,c,d (and subsequent diagrams) to indicate where the model matches the observed low-$\delta^{34}S$ of late-stage sulphides.

Motzfeldt

The magmatic and fluid evolution of Motzfeldt has been described by numerous authors[10–15]. Our study investigated the altered nepheline syenites of the Motzfeldt Sø Center (MSC),

equivalent to the SM1 classification of ref. [10]. These syenites contain a significant amount of pyrochlore and represent a low grade, large tonnage Ta-Nb deposit. Adjacent to the main ore body we identified pegmatites and veins that contained molybdenite (Fig. 4b). While other late-stage fluorite veins, injected into the surrounding rock (older syenites, granitic basement and supracrustals), contained pyrite, chalcopyrite and barite.

Pervasive late-stage alteration has taken place at MSC and this makes it difficult to place firm constraints on earlier (magmatic) phases of activity. There is a notable absence of sulphides in the MSC nepheline syenites and the mafic mineralogy is restricted to rare clusters of highly altered mica, amphibole and magnetite. Despite these challenges, ref. [13] did manage to apply two-oxide thermometry to suggest formation temperatures of ~640–690 °C at fO_2 values below the QFM buffer (ΔQFM –0.8 to –1.3).

Hematite occurs throughout the MSC[15] and provides compelling evidence that the fluid increased above the HM buffer during the final phase of evolution. This oxidation is linked to the influx of meteoric fluids, and is supported by the low-$\delta^{18}O$ and δD values for mineral separates from the MSC and meteoric-like $\delta^{18}O$ and δD of inclusion water[13]. Temperatures from fluid inclusion isotopes[13] suggest that this fluid mixing occurred around 150–300 °C, overlapping the temperatures from $\delta^{34}S$ geothermometry of chalcopyrite and barite in the late stage veins (~260 °C).

To assess $\delta^{34}S$ variations at Motzfeldt we evaluated changes in pH and fO_2 at 250 °C (i.e. a close match to the fluid inclusion temperatures and $\delta^{34}S$ geothermometry). Chalcopyrite and pyrite were found in multiple veins and so we show phase diagrams for the Fe-S-HCO_3-O and Cu-Fe-S-HCO_3-O systems (Supplementary Figure 4, note HCO_3 is included because it provides constraints on pH). We assume that the magmatic fluid was initially reduced ΔQFM = –1 (ref. [13]), and estimate an initial pH of 7. This pH value was selected because it is reasonable to assume that the fluids were comparable to Ilímaussaq[4] (above) and also because MSC fluid inclusions[13] frequently contain CO_2, requiring a pH < 8 (Supplementary Figure 4). S activity is difficult to quantify and so we varied log aS_2 between –2 and –3. The higher S activity (Supplementary Figure 4a–d) is identical to the values used for Ilímaussaq[5] (Supplementary Figure 2). Given the sparsity of S-bearing minerals at Motzfeldt and the fact that S concentrations in fluid inclusions are about an order of magnitude less than at Ilímaussaq[3,13], the lower S activity (–3, Supplementary Figure 4e–h) is likely to be more

realistic for this system. It is worth noting, however, that changing the S activity does not radically alter the mineral stability fields in these simplified systems (Supplementary Figure 4).

Trajectories of pH and fO_2 change are shown on Supplementary Figure 4f, g, h, and plotted in Figure 4b. It is evident that increasing fO_2 (oxidation), consistent with the collapse of a reduced system and influx of oxidizing fluids, can account for both the low-$\delta^{34}S$ values of sulphides (Fig. 4b), the presence of barite (SO_4 species, Supplementary Figure 4) and the abundant hematite in the MSC samples.

Ivigtût

The Ivigtût alkali granite and cryolite (Na_3AlF_6) deposit have also been the focus of decades of research[16–18]. Based on the mineralogy of the deposit three evolutionary stages are envisaged[16]. The first stage is associated with high temperatures (~500–600 °C) and begins with the injection of a mantle-derived aluminofluoride melt (exceptionally rich in F-, CO_3^{2-} and Na) into the host granite. This melt then separated into fluoride and siliceous fractions and began crystallizing siderite-quartz and siderite-cryolite. Both units are rich in sulphide minerals, in particular, chalcopyrite and galena.

In the latter stages of the deposit, ref. [16] noted lower temperatures (< 350 °C) and defined two main assemblages of fluorite-topaz and fluorite-cryolite. Scattered throughout these units are aggregates of other sodium aluminium fluorides including weberite and stenonite, as well as hydrous (OH or H_2O bearing) minerals (paragonite and jarlite), sulphides (pyrite and galena) and sulphates (barite). It was previously envisaged that these mineral assemblages may have crystallized in cavities generated by explosive activity[16]. In the final phase small pockets of secondary aluminofluorides (e.g., pachnolite, ralstonite and thomsenolite) were generated and record the last phases of mineral precipitation, likely down to temperatures of ~100 °C.

It is worth noting that our new $\delta^{34}S$ data from Ivigtût mirror the mineralogical changes, i.e. those sulphide minerals (galena, chalcopyrite and sphalerite) from the early formed high-temperature units (containing quartz, cryolite and siderite) mostly show values between –1 and 3 ‰ (Fig. 4c), while those from later low-temperature parageneses (pyrite and galena) associated with fluorite, stenonite, weberite and jarlite display a much larger, more positive range from 3 to 10 ‰ (Fig. 4c).

Fluid inclusion studies of the Ivigtût assemblages suggest that the deposit represents a zone of mixing between a mantle-derived volatile-rich fluid and a meteoric brine[18]. The saline aqueous component of the fluid inclusions have isotopic signatures ($\delta^{18}O$ and δD) and element ratios (Li/Br and Li/Cl) consistent with a meteoric source that is analogous to Canadian Shield brines[18]. Further evidence for brine mixing in the final stages of the deposit is the abundance of Ca, Mg, Sr, Ba, OH and H_2O bearing minerals (that are lacking in early, anhydrous F-dominated stage).

To evaluate S isotope fractionation due to changing pH and fO_2 in the initial magmatic fluid we generated phase diagrams (Supplementary Figure 5). Formation pressures were assumed to be 1 kbar; comparable to the other intrusions, and in the range estimated from fluid inclusions (1–1.5 kbar[18]). Thermodynamic modelling[18,19] suggests that cryolite formation takes place under acidic conditions of pH 5–6 and a temperature of 300 °C was assumed (consistent with estimates from fluid inclusion isotopes[18] and $\delta^{34}S$ geothermometry, Figure 4c). S isotope fractionation for the Fe-HCO_3-Si-S-O, Cu-Fe-HCO_3-Si-S-O and Pb-HCO_3-Si-S-O systems is shown in Supplementary Figure 5.

Our best estimate for the initial composition (asterisk, Supplementary Figure 5b, d, e) is around the QFM buffer, which is consistent with the dominance of siderite as well as the predominance of CO_2 over CH_4 in the fluid inclusions[18]. Importantly, galena and chalcopyrite, the most abundant sulphides in the Stage 1 samples (Fig. 4c), are both stable at this starting temperature-pH-fO_2 value.

Our favoured evolutionary trajectory involves oxidation of the F^- and CO_3^{2-} rich fluids and explains the low-$\delta^{34}S$ observed in a few Stage 1 sulphides (chalcopyrite and galena, Fig. 4c). Note that changes in the pH of this initial fluid (Supplementary Figure 5c) cannot explain the appearance of barite (SO_4 species, Supplementary Figure 5). Increasing fO_2 explains the occurrence of barite, chalcopyrite and galena (Supplementary Figure 5d, e) and their respective $\delta^{34}S$ (Fig. 4c). Our model also predicts stability of pyrite and hematite during oxidation (Supplementary Figure 5c). Detailed studies of the sulphide mineralogy support this[20], revealing that marcasite (a polymorph of pyrite) and hematite are commonly found replacing early-formed siderite.

The above scenario does not, however, explain the sulphide array with high-$\delta^{34}S$ (Fig. 4d). An alternative model is required, and we suggest that a suitable process would be the reduction of an infiltrating brine (with seawater-like $\delta^{34}S$). We model this process in the Fe-S-O and Pb-S-O systems, and since fluid inclusions suggest a Canadian Shield brine we use values of pH (7) and $\delta^{34}S_{\sum S}$ (20 ‰) that are typical of these fluids[21,22] and also consistent with Mesoproterozoic seawater sulphate[23]. The results are shown in Supplementary Figure 6a–d and show that the reduction of brine with high-$\delta^{34}S$, typical of seawater sulphate, can explain the high-$\delta^{34}S$ values of pyrite and galena and the presence of barite with $\delta^{34}S$ of ~21 ‰ (Fig. 4d).

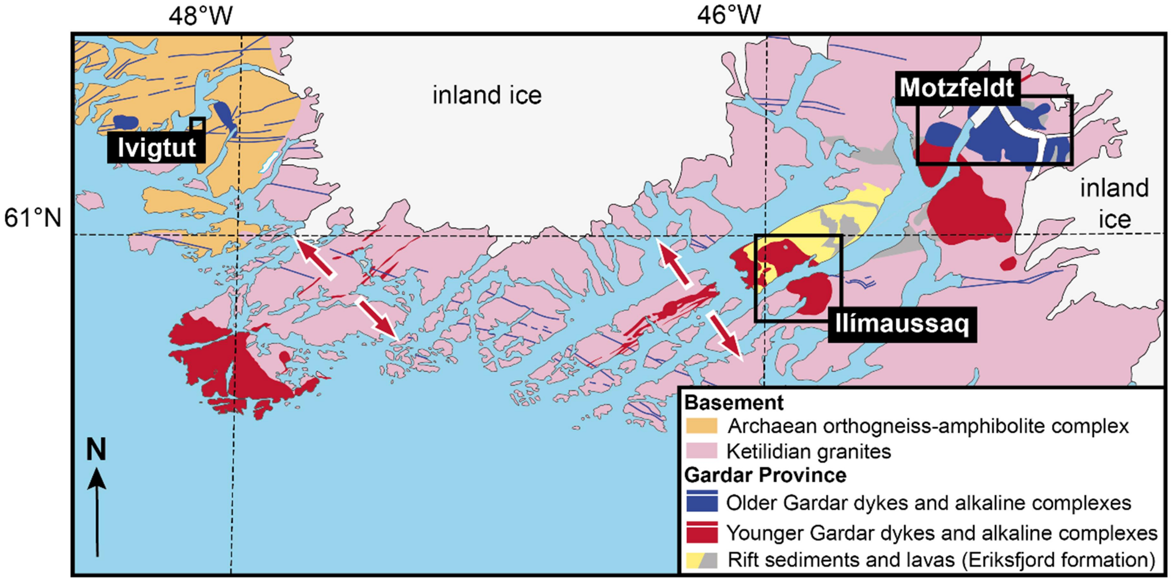

Supplementary Figure 1: Regional geological map of the Mesoproterozoic Gardar province. Geochronology suggests that Gardar magmatism took place in two phases centred around ~1280 and ~1160 Ma[24]. Motzfeldt and Ivigtût belong to the older phase of Gardar rifting and have ages of 1273 ± 8 and 1275 ± 2 Ma, respectively[14,25]. Ilímaussaq is younger with ages of 1166 ± 9 and 1160 ± 5 Ma acquired from U–Pb dating of zircon and baddeleyite[25,26]. Most Gardar intrusions, including Ilímaussaq and Motzfeldt, have been emplaced into the Ketilidian granites (~1800 Ma), although a few of the early Gardar complexes, including Ivigtût, have been emplaced into Archaean metamorphic basement (>2800 Ma). At Ilímaussaq and Motzfeldt, rift-related lavas and terrestrial sandstones comprising the Eriksfjord Formation are also found at the roof and margins of the intrusions, and unconformably overlying the Ketilidian granites.

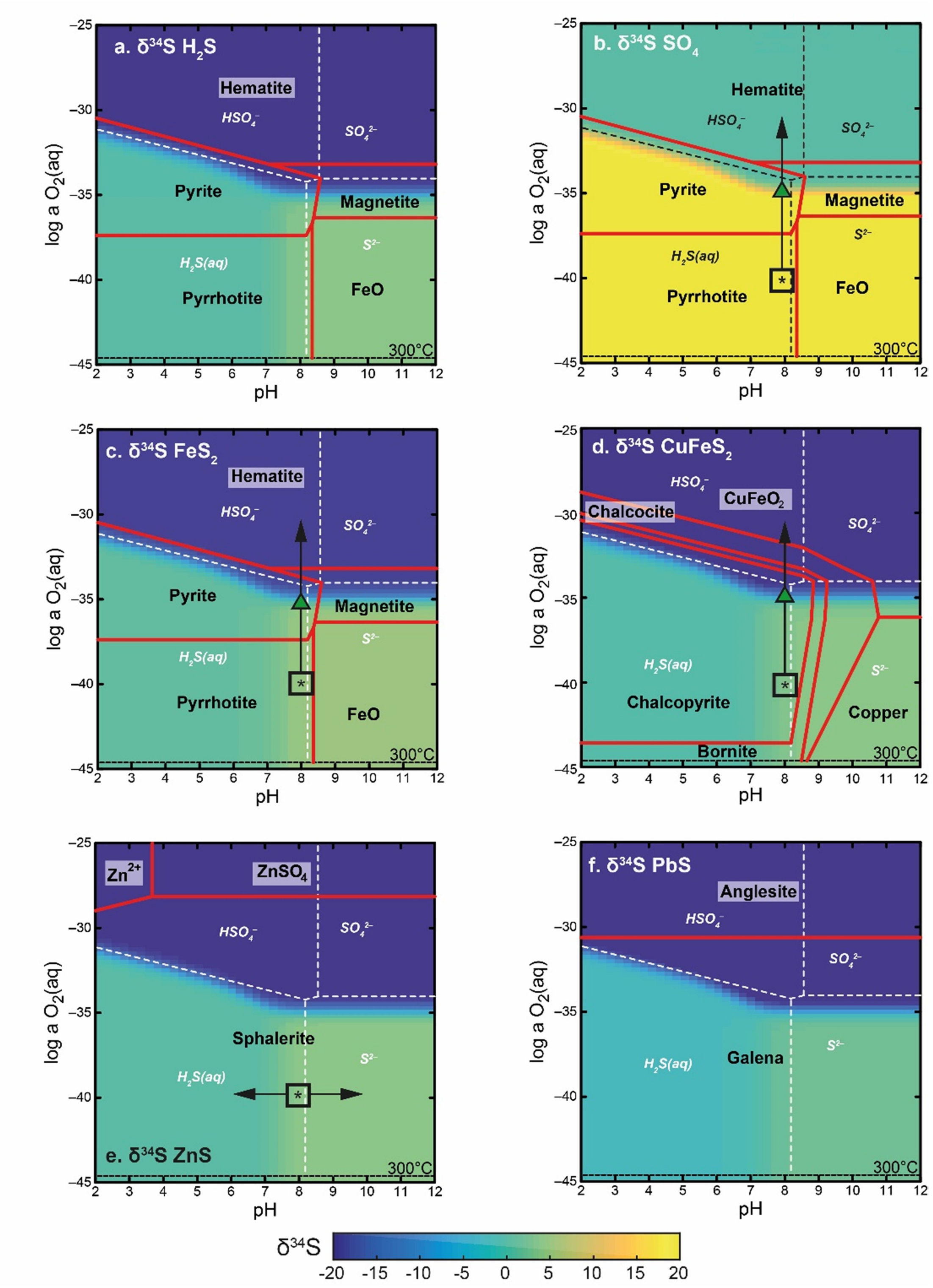

Supplementary Figure 2: pH–fO_2 diagram for magmatic-hydrothermal veins of Ilímaussaq. Stability diagrams are shown for the simplified systems Fe-S-O, Cu-Fe-S-O, Pb-S-O and Zn-S-O. The stability of the main component, Fe, Cu, Pb or Zn is shown by the thick red lines, while stability of the S phases is shown by the dashed lines. The $\delta^{34}S$ of the different S species in a–f were calculated using the methods of ref. [27] with values shown by the colour bar. $\delta^{34}S$ calculations assume: temperature = 300 °C; ionic strength = 1 (following ref. [4]); log aS_2 = –2 and $\delta^{34}S_{\Sigma S}$ = 1.8 ‰. The pH (8) and fO_2 (–40, QFM–5) of the initial magmatic fluid is shown by the asterisk (*). Arrows show pH and fO_2 trajectories for the fluid (i.e. the profile lines in Supplementary Figure 3 and Figure 4a) and the green triangle flags when the model matches the observed $\delta^{34}S$ of fenites (Figure 4a).

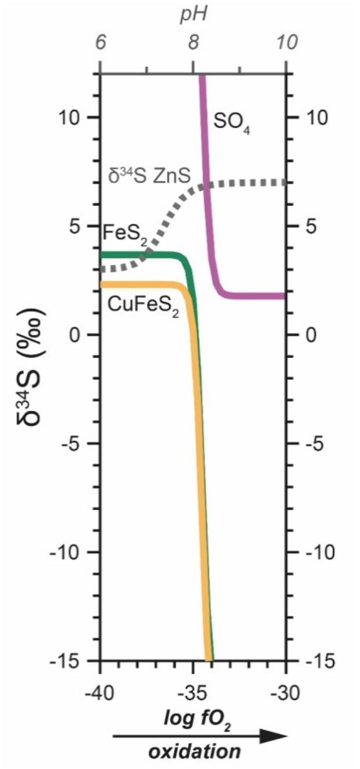

Supplementary Figure 3: Sulphur isotope fractionation caused by changing pH and fO_2. S isotope change due to varying pH is shown by the dashed grey line. While isotopic change due to variations in fO_2 is shown by the solid coloured lines. These correspond to the arrowed line profiles shown in Supplementary Figure 2.

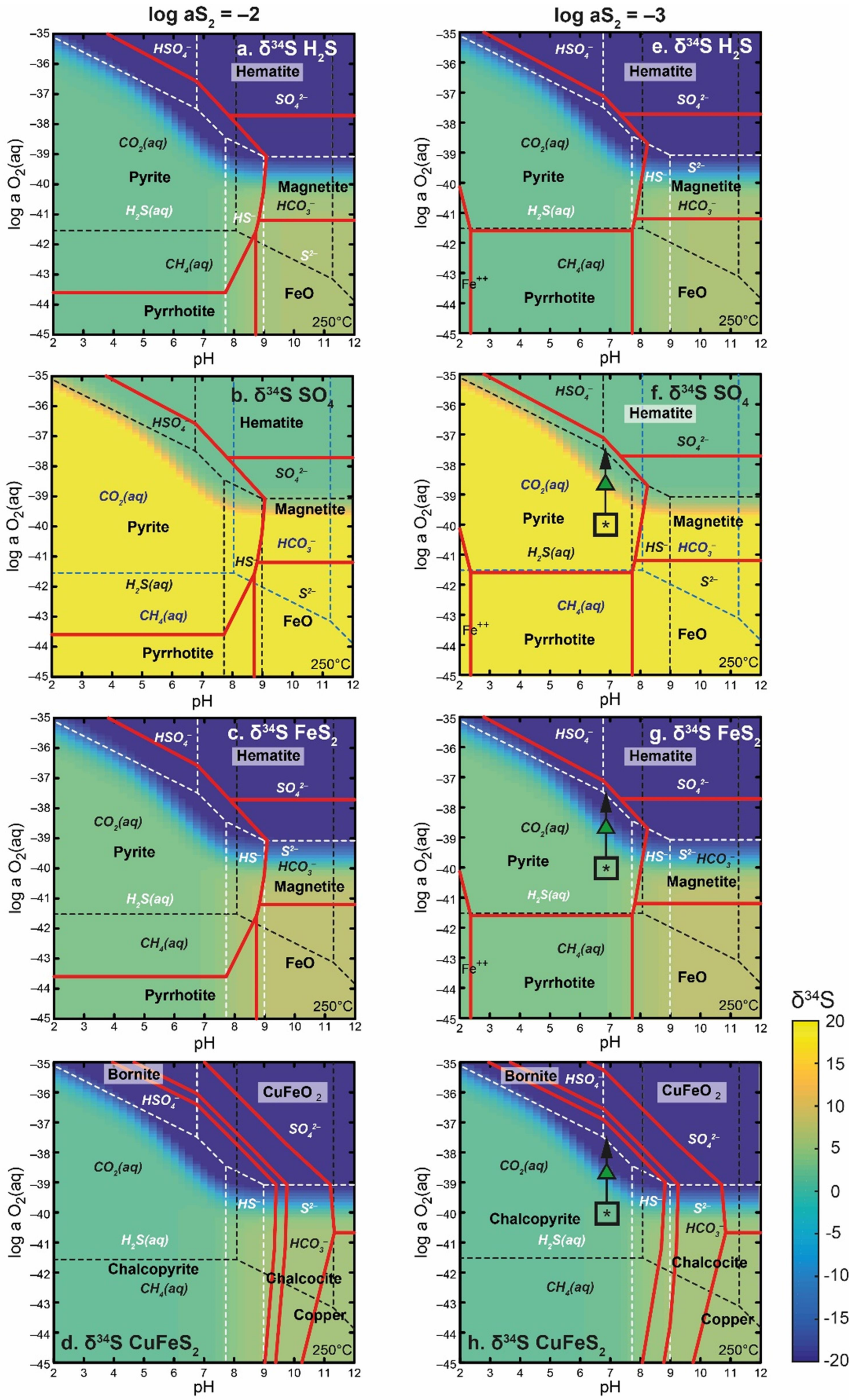

Supplementary Figure 4: pH–fO$_2$ diagram for the Motzfeldt roof zone. Stability diagrams are shown for the simplified systems Fe-S-HCO$_3$-O and Cu-Fe-S-HCO$_3$-O. The stability of the main phase, Fe or Cu, is shown by the thick red lines, while stability of S and C phases is shown by the dashed lines. The δ^{34}S of the different S species (a–h) were calculated using the methods of ref. [27] with values shown by the colour bar. δ^{34}S calculations assume: temperature = 250 °C; ionic strength = 1 and $\delta^{34}S_{\Sigma S}$ = 2 ‰. The log aS$_2$ was varied between –2 (a–d) and –3 (e–h); the latter are more realistic for Motzfeldt (see text). The pH (7) and fO$_2$ (–40, QFM–1) of the initial magmatic fluid is shown by the asterisk (*). Arrows show pH and fO$_2$ trajectories for the fluid (i.e. the profile lines in Fig. 4b) and the green triangle flags when the model matches the observed δ^{34}S of vein sulphides and sulphates (Fig. 4b).

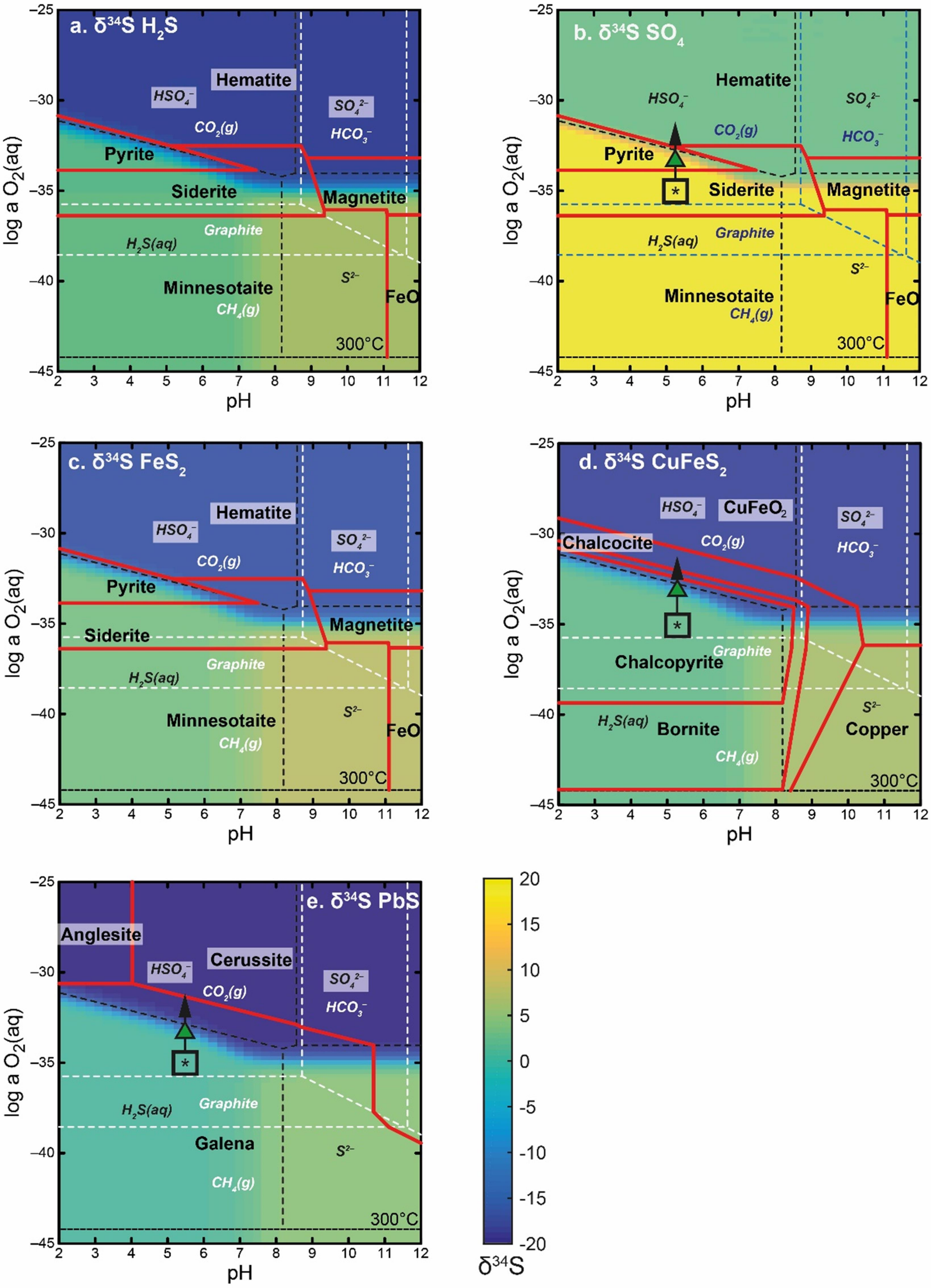

Supplementary Figure 5: pH– fO_2 diagrams for the Ivigtût cryolite deposit. Stability diagrams are shown for the simplified systems Fe-HCO_3-Si-S-O, Cu-Fe-S-HCO_3-Si-O and Pb-S-HCO_3-Si-O. The stability of the main component, Fe, Pb or Zn is shown by the thick red lines, while stability of the S and C phases is shown by the dashed black and white/blue lines, respectively. The $\delta^{34}S$ of the different S species (a–e) were calculated using the methods of ref. [27] with values shown by the colour bar. $\delta^{34}S$ calculations assume: temperature = 300 °C; ionic strength = 1; log aS_2 = –2.5 and $\delta^{34}S_{\Sigma S}$ = 2.5 ‰. The pH (5.5) and fO_2 (–35, ~QFM) of the initial reduced magmatic fluid is shown by the asterisk (*). Arrows show pH and fO_2 trajectories for the fluid (note that the profile lines for the fO_2 changes are shown in Fig. 4c) and the green triangle flags when the model matches the observed lowest $\delta^{34}S$ of Stage 1 sulphides (Fig. 4c).

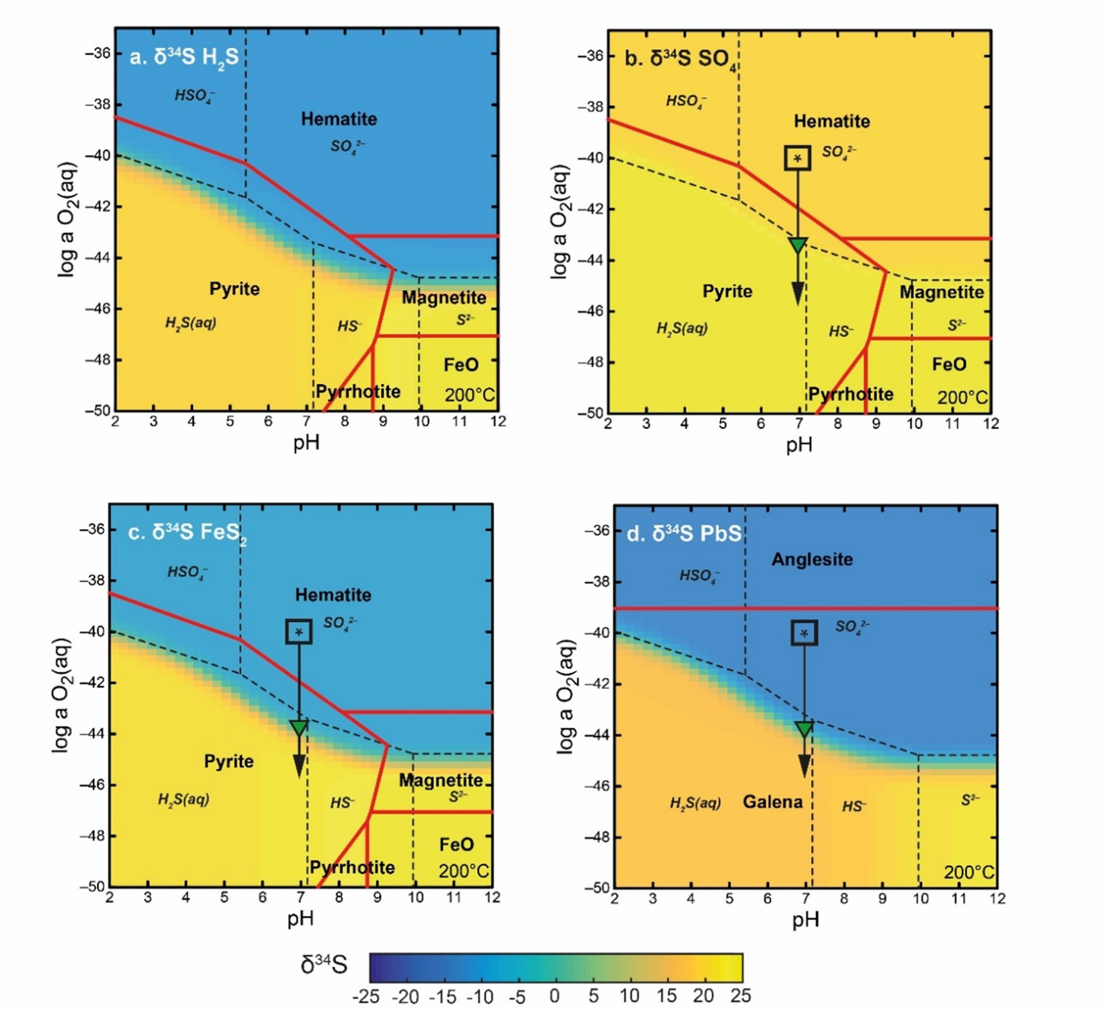

Supplementary Figure 6: pH– fO_2 diagram for a sulphate-rich crustal brine interacting with the Ivigtût magmatic body. Stability diagrams are shown for the simplified systems Fe-S-O and Pb-S-O. The stability of the main component Fe or Pb is shown by the thick red lines, while stability of the S phases is shown by the dashed lines. The $\delta^{34}S$ of the different S species (a–d) were calculated using the methods of ref. [27] with values shown by the colour bar. $\delta^{34}S$ calculations assume: temperature = 200 °C; ionic strength = 1; log aS_2 = –2 and $\delta^{34}S_{\Sigma S}$ = 20 ‰. The initial pH (7) and fO_2 (–40, ~HM) of the initial oxidised fluid, representative of a Canadian Shield brine, is shown by the asterisk (*). Arrows show the fO_2 trajectories for the fluid (the profile line is shown in Fig. 4d) and the green triangle flags when the model matches the observed high-$\delta^{34}S$ of the final stage sulphides (Fig. 4d).

SUPPLEMENTARY REFERENCES

1. Marks, M. A. W. & Markl, G. The Ilímaussaq alkaline complex, South Greenland. in *Layered Intrusions* 649–691 (Springer, 2015).
2. Konnerup-Madsen, J. & Rose-Hansen, J. Composition and significance of fluid inclusions in the Ilímaussaq peralkaline granite, south Greenland. *Schweizerische Mineral. und Petrogr. Mitteilungen* **107**, 317–326 (1984).
3. Graser, G., Potter, J., Köhler, J. & Markl, G. Isotope, major, minor and trace element geochemistry of late-magmatic fluids in the peralkaline Ilímaussaq intrusion, South Greenland. *Lithos* **106**, 207–221 (2008).
4. Markl, G. & Baumgartner, L. pH changes in peralkaline late-magmatic fluids. *Contrib. to Mineral. Petrol.* **144**, 331–346 (2002).
5. Hettmann, K. *et al.* The geochemistry of Tl and its isotopes during magmatic and hydrothermal processes: The peralkaline Ilimaussaq complex, southwest Greenland. *Chem. Geol.* **366**, 1–13 (2014).
6. Markl, G., Marks, M. A. W., Schwinn, G. & Sommer, H. Phase Equilibrium Constraints on Intensive Crystallization Parameters of the Ilimaussaq Complex, South Greenland. *J. Petrol.* **42**, 2231–2257 (2001).
7. Karup-Møller, S. *The ore minerals of the Ilímaussaq intrusion: their mode of occurrence and their conditions of formation*. (Grønlands geologiske undersøgelse, 1978).
8. Babiel, R., Marks, M. A. W., Neumann, U. & Markl, G. Sulfides in alkaline and peralkaline rocks: textural appearance and compositional variations. *Neues Jahrb. für Mineral. - Abhandlungen J. Mineral. Geochemistry* **195**, 155–175 (2018).
9. Graser, G. & Markl, G. Ca-rich ilvaite-epidote-hydrogarnet endoskarns: A record of late-magmatic fluid influx into the persodic Ilímaussaq complex, South Greenland. *J. Petrol.* **49**, 239–265 (2008).
10. Emeleus, C. H. & Harry, W. T. *The Igaliko-nepheline-syenite-complex, south Greenland*. (Reitzel, 1970).
11. Jones, A. P. & Larsen, L. M. Geochemistry and REE minerals of nepheline syenites from the Motzfeldt Centre, South Greenland. *Am. Mineral.* **70**, 1087–1100 (1985).
12. Jones, A. P. Mafic silicates from the nepheline syenites of the Motzfeldt centre, South Greenland. *Mineral. Mag.* **48**, 1–12 (1984).
13. Schönenberger, J. & Markl, G. The magmatic and fluid evolution of the motzfeldt intrusion in South Greenland: Insights into the formation of agpaitic and miaskitic

rocks. *J. Petrol.* **49**, 1549–1577 (2008).
14. McCreath, J. A., Finch, A. A., Simonsen, S. L., Donaldson, C. H. & Armour-Brown, A. Independent ages of magmatic and hydrothermal activity in alkaline igneous rocks: The Motzfeldt Centre, Gardar Province, South Greenland. *Contrib. to Mineral. Petrol.* **163**, 967–982 (2012).
15. McCreath, J. A., Finch, A. A., Herd, D. A. & Armour-Brown, A. Geochemistry of pyrochlore minerals from the motzfeldt center, south greenland: The mineralogy of a syenite-hosted Ta, Nb deposit. *Am. Mineral.* **98**, 426–438 (2013).
16. Pauly, H. & Bailey, J. C. Genesis and evolution of the Igvitut cryolite deposit, SW Greenland. *Meddelser om Grønland, Geosci.* (1999).
17. Goodenough, K. M., Upton, B. G. J. & Ellam, R. M. Geochemical evolution of the Ivigtut granite, South Greenland: A fluorine-rich 'A-type' intrusion. *Lithos* **51**, 205–221 (2000).
18. Köhler, J., Konnerup-Madsen, J. & Markl, G. Fluid geochemistry in the Ivigtut cryolite deposit, South Greenland. *Lithos* **103**, 369–392 (2008).
19. Prokof'ev, V. B., Naumov, V. B., Ivanova, G. F. & Savel'eva, N. I. Fluid inclusion studies in cryolite and siderite of the Ivigtut deposit (Greenland). *Neues Jahrb. für Mineral. Monatshefte* **1**, 32–38 (1991).
20. Soen, O. I. & Pauly, H. A sulphide paragenesis with pyrrhotite and marcasite in the siderite-cryolite ore of Ivigtut, South Greenland. *Meddelelser om Grønl.* **175**, 1–55 (1967).
21. Bottomley, D. J., Conrad Gregoire, D. & Raven, K. G. Saline ground waters and brines in the Canadian Shield: Geochemical and isotopic evidence for a residual evaporite brine component. *Geochim. Cosmochim. Acta* **58**, 1483–1498 (1994).
22. Fritz, P., Frape, S. K., Drimmie, R. J., Appleyard, E. C. & Hattori, K. Sulfate in brines in the crystalline rocks of the Canadian shield. *Geochim. Cosmochim. Acta* **58**, 57–65 (1994).
23. Farquhar, J., Nanping, W. U., Canfield, D. E. & Oduro, H. Connections between sulfur cycle evolution, sulfur isotopes, sediments and base metal sulfide deposits. *Econ. Geol.* **105**, 509–533 (2010).
24. Upton, B. G. J. *Tectono-magmatic evolution of the younger Gardar southern rift, South Greenland. Geological Survey of Denmark and Greenland Bulletin* (2013).
25. Upton, B. G. J., Emeleus, C. H., Heaman, L. M., Goodenough, K. M. & Finch, A. A. Magmatism of the mid-Proterozoic Gardar Province, South Greenland: Chronology,

petrogenesis and geological setting. *Lithos* **68**, 43–65 (2003).

26. Krumrei, T. V., Villa, I. M., Marks, M. A. W. & Markl, G. A 40Ar/39Ar and U/Pb isotopic study of the Ilímaussaq complex, South Greenland: Implications for the 40K decay constant and for the duration of magmatic activity in a peralkaline complex. *Chem. Geol.* **227**, 258–273 (2006).
27. Ohmoto, H. Systematics of sulfur and carbon isotopes in hydrothermal ore deposits. *Econ. Geol.* **67**, 551–578 (1972).
