# Supplementary material for: Sulphur isotopes of alkaline magmas unlock long-term records of crustal recycling on Earth
Source: Nat Commun. 2019 Sep 16;10:4208. doi: 10.1038/s41467-019-12218-1 (PMC6746797; doi:10.1038/s41467-019-12218-1)
Supplement: Supplementary file 3 — Description of Additional Supplementary Files [file 41467_2019_12218_MOESM3_ESM.docx]

**Description of Supplementary Files**

**File Name: Supplementary Data 1**

**Description:** Sulphur isotope results for mineral separates from the Gardar Province.

**File Name: Supplementary Data 2**

**Description:** Sulphur concentration and isotope results for whole-rock samples from the Gardar Province. Note that we extracted sulphide phases as Ag2S before isotopic analysis.

**File Name: Supplementary Data 3**

**Description:** Compilation of sulphur isotopes from alkaline and carbonatite complexes worldwide. Data are ordered by age (with youngest first).
