# Supplementary material for: Sulphur isotopes of alkaline magmas unlock long-term records of crustal recycling on Earth
Source: Nat Commun. 2019 Sep 16;10:4208. doi: 10.1038/s41467-019-12218-1 (PMC6746797; doi:10.1038/s41467-019-12218-1)
Supplement: Supplementary file 2 — Peer Review File [file 41467_2019_12218_MOESM2_ESM.pdf]

Reviewers' comments:

Reviewer #1 (Remarks to the Author):

This paper presents sulfur isotopes compositions of sulfur-bearing phases from three alkaline intrusions in Greenland, as well as a global compilation of literature data from alkaline intrusions elsewhere. This database spans most of the Earth's history and allows to detect secular variations in the sulfur isotope composition of the Earth's mantle, as well as comparison with surface reservoirs. The authors find a distinct decrease in d34S in the Pahnerozoic, which they ascribe to sulfur subduction in the Archean and recycled back to the surface ca. 1.5 billion years later.

This is a thought-provoking and well-written paper of broad interest, and I recommend publication with only some minor revisions.

There are a few points that may need some clarification.

- It isn't quite made clear what the Greenland data adds to the already quite comprehensive database, as it doesn't seem to fill a time gap. It appears it is the detailed evaluation of sulfur fractionation processes, but it could be phrased more explicitly.

- One of the goals mentioned is to explore whether d34S can be used to probe REE mineralisation. However, this is only very casually mentioned in the remainder of the manuscript, and I don't think the conclusions in the 'Implications and Conclusions' section are substantiated elsewhere in the text. This part needs to be either strengthened or removed.

- Although the authors do a good job of looking at sulfur fractionation processes during magma evolution, they say little about crustal contamination. Perhaps it was limited but can it be ignored?

- On face value, the correlation in Fig. 4 between the drop in d34S in both sediments and the mantle is more suggestive of a direct link than of the authors' interpretation (that they are decoupled, the mantle value being the result of subduction a billion years earlier). Can the source of alkaline magmas have been modified by more recent subduction fluids of early Phanerozoic sediments? There's a few 100 Ma even for the Kola samples to do this. Worth discussing further if only to show the alkaline magmas are from areas far from any sutures/former subduction zones.

- In Fig. 2 I am confused where the x-axis data (T, pH and fO2) come from, are these measured, modelled or calculated? It should be mentioned in the caption.

- In Fig.4 the two different scales are confusing, and the fact that all mantle source values appear to fall right in-between the sediment sulfide and seawater average is fortuitous (or perhaps intentional) based on the relative scaling. It would be better in my opinion to plot the data on the same scale,

and then have a magnified d34S plot with the alkaline S record above or below in a separate part of the figure.

Cees-Jan de Hoog

University of Edinburgh

Reviewer #2 (Remarks to the Author):

This is a paper with a split personality. Most of it (≈4/5) is an in-depth discussion of how igneous processes associated with the generation, emplacement, and late-stage evolution of alkaline magmas lead to predictable 34S-32S fractionations. The rest of it (≈1/5) is a speculative link between the temporal evolution of the inferred d34S values for the source of alkaline magmas, and the d34S record of sedimentary sulfides through time. While both parts of this study are new, the majority of the claims discussed in this paper would not really be of interest to the broad readership of Nature Communications. The evolution of alkaline magmas, including their associated mineralization histories, is a topic that is more appropriate for a specialist audience. The claim that there is a temporal link between mantle sources for alkaline magmas and the surface sulfur cycle, and that this link constrains the style and rate of surface-mantle material transfer, is a topic that potentially reaches across the spectrum of the Nature Communications. As it currently stands however I don't think that manuscript describes, explores, or justifies this link well enough to be published in Nature Communications. In what follows I provide some general feedback that may help the authors consider how to revise a manuscript that focuses more on the novel suggestions in the paper. I am happy to be contacted about this review at boswell.wing@colorado.edu - Boz Wing

(1) How were the 'source' d34S values for alkaline magmas estimated?

To my mind this represents the most natural place for a link between the detailed modelling in the first parts of the paper, and the speculations later in the paper. That is, was the modelling in the first part of the paper run in an 'inverse' sense such that the full suite of measurements could be brought to bear on the d34S estimates for the source? This estimation procedure should be described in more detail and uncertainties should be reported on the inferred d34S values of the sources.

(2) Is a mantle circulation 'time lag' for recycling of surface S into the mantle source for alkaline magmas the only hypothesis that can explain the data?

While I am pretty much convinced that Proterozoic alkaline magmas have have S sources with consistently slightly positive d34S values, and Phanerozoic alkaline magmas have S sources with both slightly positive and slightly negative d34S values, the manuscript does not convince me that recycling of ancient surface S is the most viable explanation for this pattern. The 'gold standard' observation that would buttress this speculation would be observation of non-negative D33S values in the Proterozoic S sources, which would imply that the 1-1.5 Gyr circulation timelag has been in place for much of Earth history.

Barring that, a revised manuscript should consider alternate hypotheses and attempt to rule them out. For example, I can think of two hypotheses that might explain the data, but might not be as impactful as the one favored in the present version of the manuscript. The first is local in space - have the alkaline magmas simply inherited their S isotope compositions from local host rocks? I know the authors have the expertise to address this. The second is local in time - is the S source to the alkaline magmas simply roughly the same age as the magmas themselves? That is, instead of Gyr time lags in supply of surface S to the mantle source regions of alkaline magmas, can the data rule out 100 Myr time lags? In addition to these two hypotheses, I can also speculate a more starry-eyed one. The modelling illustrates an exquisite sensitivity of d34S values in these systems to f_O2 variations. Evidence for secular f_O2 variations in the mantle has been a 'holy grail' for Earth historians for a long time - can the d34S identified here be explained by temporal changes in f_O2 in the mantle source regions for alkaline magmas? From my non-expert perspective, one might be able to argue that small degree partial melts like alkaline magmas might be the right place to look for f_O2 variations rather that the historical focus on more extensive mantle melts like basalts and their siblings.

From a similar perspective, the current manuscript bases its speculation on something that is itself speculative. The hypothesis that there is a missing 34S-depleted reservoir from Earth's surface in the Proterozoic is a model-based inference. As far as I know it has not been verified with actual data, and the variability in the d34S record in pyrites through time looks like it might also support other hypotheses (for example, limited sulfate supply to the regions of pyrite formation).

(3) Despite promises, this comment is about the first 4/5 of the current manuscript. The S isotopic modelling (and Fig 2 in particular) does not make it clear which results represent dependent variables, and which represent independent variables. My read is that the d34S values are measured independent variables, and the things like pH, f_O2, and T are all dependent variables. However this relationship is obscured by the choice to plot actual data in figure 2, with d34S values on the y-axis and pH, f_O2, and T on the x-axis. This is fine to do for model calculations, but gives the misleading impression that precise values for pH, f_O2, and T have been measured for each sample with a measured d34S value.

Minor comment: the manuscript repeatedly refers to the "Precambrian" and to "Precambrian sulfur". More than almost any other geochemical quantity, however, Precambrian S is divisible between Archean and Proterozoic S. Making this distinction clear, I think, will go a long ways toward strengthening any interpretations made in a revised manuscript.

Reviewer #3 (Remarks to the Author):

Here is a review of Sulphur isotopes of alkaline magmas support deep recycling of Earth's crust

By Hutchison and co-authors.

In this study, the authors report on the S isotope composition of granites and syenitic rocks from Greenland. In the following review, I'll refer to these as simply 'granites' out of clarity. The author also present a compilation of d34S data for other granites. They state that granites of different ages can record the composition of OIB mantle sources at different times (after some modeling). They report a d34S pattern that they suggest mimics the d34S evolution of sediments. They suggest that this is a record of interaction of surface and the mantle. Although the article is well redacted, multiple flaws are present:

1:

The authors describe how complex their system is: their highly evolved melts are granites. These are formed at low temperature, interact with fluids, and the authors have to invoke various pH of the fluids to derive the composition of the parental magma, noted $\delta 34S_{\sum S}$ by the authors. Although the authors state that $\delta 34S_{\sum S}$ is a mantle source estimate (L. 217), it is only a parental melt estimate.

From measured d34S values scattering between -1 and +4‰, the authors suggest parental melts compositions of 0, 2.0 and 2.5‰ for their three systems. Note that no uncertainty is offered for these estimates. These estimate relies on a isotope mass balance in the H2S-HS-S2- system in fluids at temperatures between 600 and 200 C. Discussion of how the parental melts could have acquired these three distinct d34S values is not offered in the detail. Instead, the authors assume that these values represent mantle sources – with no discussion offered, or citations given. Fractionating sulfides are known to occur during partial melting and early in the differentiation. The d34S effect is likely insignificant in basalts (No citation offered here) but unknown in lower-T melts.

2.

As said by the authors themselves, "d34S is exceptionally sensitive to low temperature oxidation" (L 217) but it also very sensitive to crustal assimilation. Granite-derived samples typically don't show significant S concentrations (< 10 ppm S). This is because sulfur was lost by sulfide segregation earlier in the differentiation sequence. The authors don't present the S contents of their melts, but the occurrence of any S is telltale of crustal assimilation or S inherited by crustal melting. Note that melts going through continental crust are well described to assimilate contaminants and drastically overprint the S isotope composition of the melts (Bekker et al. 2009 in science is one example among others – not cited)

In fact, based on the data presented in this study, the authors cannot rule out the following suggestion:

Melts that went through the Proterozoic crust are biased towards positive d34S because they have assimilated Proterozoic sediments (or contemporary S-bearing metasomatizing fluids) with positive d34S. Conversely, negative d34S observed elsewhere in phanerozoic settings are due to the assimilation of phanerozoic sediments (or phanerozoic S-bearing metasomatizing fluids) before emplacement of the melts. This is why the d34S record of alkaline melts mimics the d34S record of sediments.

3.

Instead of discussing crustal assimilation, the authors assign their d34S estimate of parental melts to mantle sources. They present the idea that negative d34S values in phanerozoic granites are accounted for by the contribution of subducted components carrying negative d34S that have a Proterozoic age. Note that such components are not observed in the sedimentary record but have been invoked to be lost to the mantle by subduction to satisfy a surface mass balance

If the authors claim is correct, it seems to undermine their suggestion that d34S pattern in alkaline melts mimics the sedimentary record: The coinciding low d34S value in phanerozoic granites and sediments becomes fortuitous, since according to the authors themselves, granite values would be due to recycling of Proterozoic components with negative d34S, while sedimentary low d34S values are accounted for by the increase of sulfate content in the oceans after roughly 550 Ma.

4.

Finally, I note that the authors have ignored studies that contradict their suggestion: The Samoa and Discovery hotspots (both of EM type) have d34S values consistent with the contribution of proterozoic sediments with positive d34S (+10‰) (Labidi et al. 2013, Nature – not cited and not shown in their fig. 4, and Labidi et al. 2015, EPSL – cited but not shown in their figure 4). Not only this contradicts the idea of negative d34S recycled in the mantle, it also shows that mantle storage of old components contributing to present-day volcanism does not lead to a d34S pattern that

mimics the surface record. The authors claim of subduction of Proterozoic sediments with negative d34S is challenged by this literature.

Furthermore, one article by Beaudy et al. (2018, Nature Comm, not cited) shows that in fact, low d34S values in canaries magma are due to sulfur degassing. This hotspot is known to show recent recycled oceanic crust (Halliday et al. 1992,1993, Thirlwall, 1997). After reconstruction, Beaudry et al show that the mantle source of this hotspots include recycled components with positive d34S. This shows that even subduction of modern sediments do not meet the prediction made by Hutchison et al.

This literature seems to rule out the claims articulated by Hutchison et al.

Reviewer's comments: italics (11pt)

Responses: plain text (11 pt)

Reviewer #1

It isn't quite made clear what the Greenland data adds to the already quite comprehensive database, as it doesn't seem to fill a time gap. It appears it is the detailed evaluation of sulfur fractionation processes, but it could be phrased more explicitly.

We agree with reviewer. The purpose of studying the Gardar Province (Greenland) was not to fill a time gap but rather to build up a detailed understanding of S isotope fractionation from source to surface (in a well-studied, well-exposed rift zone).

We have clarified this in lines 69–73: ‘While there have been a large number of S isotope investigations of alkaline complexes (Fig. 1), few[8–11] have thoroughly investigated how processes such as crustal contamination, degassing and fluid evolution (i.e. changes in temperature-pH-fO_2) altered the primary mantle signature. Understanding these processes is critical to unlocking the alkaline record of magma sources and placing these observations within the context of the global S cycle.’

Also in lines 295–300 ‘…this provides strong evidence that evolved alkaline rocks constrain magma source $\delta^{34}S$. … Only with our complete data set from multi-phase alkaline intrusions and their associated primitive magmas has it been possible to verify fully this hypothesis.’

One of the goals mentioned is to explore whether d34S can be used to probe REE mineralisation. However, this is only very casually mentioned in the remainder of the manuscript, and I don't think the conclusions in the 'Implications and Conclusions' section are substantiated elsewhere in the text. This part needs to be either strengthened or removed.

We have removed this section of the text and focused on the key finding of this paper (i.e. the S isotope record of alkaline magma sources)

Although the authors do a good job of looking at sulfur fractionation processes during magma evolution, they say little about crustal contamination. Perhaps it was limited but can it be ignored?

The issue of crustal contamination was picked up by all reviewers and we have addressed this with the addition of new data and text.

For the Gardar case study we generated new S concentration and $\delta^{34}S$ for both Gardar magmatic rocks and local crustal lithologies. This data is presented in Table 1 along with various scenarios of crustal melting and assimilation. The key discussion of crustal assimilation is provided in lines 173–189.

In short, S concentrations in the local crust are very low and when we consider various scenarios of crustal assimilation we find that even extreme crustal assimilation (50 %) have limited impact on melt $\delta^{34}S$ (generally <0.5 ‰). Such extreme scenarios are not supported by previous isotopic or petrographic studies from the Gardar (see papers cited in lines 187). Thus, significant crustal interactions can also be ruled out.

It is also very unlikely that crustal assimilation impacted $\delta^{34}S$ in our global compilation (Fig. 5, 6). While granites and mafic lavas can have S concentrations <10 ppm (making them very susceptible to crustal assimilation, as pointed out by Reviewer 3), S concentrations in alkaline rocks are much greater. Carbonatites have average values of ~6000 ppm (line 61) and in our sample suite S concentrations are typically several 100 to 1000 ppm (Table 1).

The fact that all studies compiled for Figures 5 and 6 report visible S minerals support high S concentrations (>>100 ppm) and makes it unlikely that their S contents have been modified by crustal interactions. Radiogenic isotope studies (cited in line 379) strongly support this, and rule out assimilation of local crust in Kola and APIP, the two regions with anomalous low-δ^{34}S. As has been emphasised by previous authors, and in our study (lines 58–60) 'The advantage of alkaline rocks, compared to more common basaltic or granitic suites, is that their low viscosities, densities and temperatures promote rapid rise to the surface which minimizes crustal interactions and preserves the mantle signature[1].'

On face value, the correlation in Fig. 4 between the drop in d34S in both sediments and the mantle is more suggestive of a direct link than of the authors' interpretation (that they are decoupled, the mantle value being the result of subduction a billion years earlier). Can the source of alkaline magmas have been modified by more recent subduction fluids of early Phanerozoic sediments? There's a few 100 Ma even for the Kola samples to do this. Worth discussing further if only to show the alkaline magmas are from areas far from any sutures/former subduction zones.

This is a very helpful suggestion and we have now included a much more detailed discussion of the links between the sedimentary and igneous δ^{34}S records (lines 405–441). We consider three scenarios (based on the reviewers' suggestions) that might account for the time-evolving trends and have added a new figure (Fig. 7) where these scenarios are clearly summarised.

The reviewer's suggestion that some of the Phanerozoic alkaline sources (e.g. Kola and APIP) could have been modified low-δ^{34}S sedimentary rocks subducted in the Phanerozoic is one of the key hypotheses considered (lines 410–415).

As the reviewer suggested we investigated whether there were any Phanerozoic sutures (former subduction zones) in the vicinity of the APIP and Kola. In both cases the nearest suture zones are Proterozoic and so we can rule out Phanerozoic subduction-related source modification (lines 424–428) and look to other hypotheses to explain their low-δ^{34}S.

In Fig. 2 I am confused where the x-axis data (T, pH and fO2) come from, are these measured, modelled or calculated? It should be mentioned in the caption.

The x-axis data are modelled and we have updated the axis of the plot to clearly state this (note Fig. 2 is now Fig. 4). We have also added a new figure (Fig. 2) where all new δ^{34}S data are plotted in histograms. This will help the reader by clearly separating the data (Fig. 2) from model interpretations (Fig. 3).

In Fig.4 the two different scales are confusing, and the fact that all mantle source values appear to fall right in-between the sediment sulfide and seawater average is fortuitous (or perhaps intentional) based on the relative scaling. It would be better in my opinion to plot the data on the same scale, and then have a magnified d34S plot with the alkaline S record above or below in a separate part of the figure.

To avoid confusion over the correct y-axis we have now plotted the igneous and sedimentary δ^{34}S in two separate panels (now Figure 6). We tried changing the y-axis scale of the igneous record as suggested by the reviewer. However, as the igneous record reflects mantle S (around 0 ‰) plus a component of sedimentary S the shifts in δ^{34}S will be much more subtle. Plotting the igneous and sedimentary δ^{34}S on the same y-axis scale makes it difficult to appreciate these subtle but real variations in igneous δ^{34}S.

To address the reviewers point we have added a box inset to the sedimentary $\delta^{34}S$ record (Fig. 6b) which shows the y-scale of the igneous $\delta^{34}S$ record (Fig. 6a). This makes it clear that the variations in sedimentary $\delta^{34}S$ are greater than those of the igneous $\delta^{34}S$ and also allows the reader to appreciate the real variations in magma source $\delta^{34}S$ (Fig. 6a).

Reviewer #2

(1) How were the 'source' d34S values for alkaline magmas estimated?

To my mind this represents the most natural place for a link between the detailed modelling in the first parts of the paper, and the speculations later in the paper. That is, was the modelling in the first part of the paper run in an 'inverse' sense such that the full suite of measurements could be brought to bear on the d34S estimates for the source? This estimation procedure should be described in more detail and uncertainties should be reported on the inferred d34S values of the sources.

We now include a paragraph that details how the magma source estimates were made and their associated uncertainties. Lines 345–355:

'Our $\delta^{34}S$ observations alongside previous studies[9] emphasise that S-rich alkaline rocks can only be used to evaluate magma source $\delta^{34}S$ when they are dominated (>90 %) by either sulphide or sulphate. For each system we averaged $\delta^{34}S$ in the most primitive, high temperature (>>300 °C) phases (mostly magmatic cumulate). When the $\delta^{34}S$ of multiple sulphide minerals was reported we only include the most reduced phase (taking pyrrhotite over pyrite) and apply temperature corrections similar to our Gardar case study (Fig. 4a–c). Well-constrained source values (Fig. 5) were only calculated for systems that met these criteria and where petrological observations confirm that the mineralogy was dominated by reduced or oxidised S only. Based on our observations from the Gardar, where early-formed sulphides are within ~1 ‰ of primitive samples, we assume that this holds globally and that magma source $\delta^{34}S$ is within ±1.5 ‰ of its early-formed S mineral value.'

While we agree with reviewer's suggestion that it would be good to use the modelling in a 'inverse' sense to evaluate magma source $\delta^{34}S$ the reality is that most alkaline systems do not have solid constraints on temperature-pH-fO_2 and this prevents us from undertaking the high-quality modelling as in our Greenland case study.

We specifically selected the Gardar region because these parameters (temperature-pH-fO_2) are well-known and also because a wide spectrum of magmatic units are exceptionally well-exposed (i.e. alkaline intrusions can be sampled over several km in the vertical, and primitive mafic dykes and diatremes can also be accessed).

For the revised manuscript we analysed these primitive dykes and diatremes because they provide the most direct samples of the Gardar mantle source (see lines 105–107). We also analysed magmatic cumulate and dykes from the alkaline intrusions; rocks that are parental to the sulphide-dominated rocks from the alkaline intrusions (Fig. 2b,d,f). All of these $\delta^{34}S$ values are positive and overlapping and as we point out in lines 292–296 that:

'…because the $\delta^{34}S$ source values calculated for early-stage sulphide-dominated rocks from the alkaline intrusions (Fig. 4a–c) are within ~1 ‰ of their parental magmatic units (Fig. 2), and also overlap the $\delta^{34}S$ of primitive samples; this provides strong evidence that evolved alkaline rocks constrain magma source $\delta^{34}S$.'

Our detailed case study demonstrates that as long as the S mineral assemblage is dominated by either reduced or oxidised S species then it is reasonable to use mineral $\delta^{34}S$ to evaluate source $\delta^{34}S$. This point has been made previously, but as we state in lines 299-

300, ‘Only with our complete data set from multi-phase alkaline intrusions and their associated primitive magmas has it been possible to verify fully this hypothesis.’

(2) Is a mantle circulation 'time lag' for recycling of surface S into the mantle source for alkaline magmas the only hypothesis that can explain the data?

We have now set up three alternate hypotheses (based on the reviewers’ suggestions) that might explain the temporal variation in magma source $\delta^{34}S$. These hypotheses are: 1) crustal contamination and a changing crustal $\delta^{34}S$; 2) changing $\delta^{34}S$ of the SCLM on ~10–100 Ma timescales and 3) deep crustal recycling via mantle plumes on ~1 Ga timescales. These are summarised in lines 405-418 and in the new Figure 7.

While I am pretty much convinced that Proterozoic alkaline magmas have have S sources with consistently slightly positive d34S values, and Phanerozoic alkaline magmas have S sources with both slightly positive and slightly negative d34S values, the manuscript does not convince me that recycling of ancient surface S is the most viable explanation for this pattern. The 'gold standard' observation that would buttress this speculation would be observation of non-negative D33S values in the Proterozoic S sources, which would imply that the 1-1.5 Gyr circulation timelag has been in place for much of Earth history.

Barring that, a revised manuscript should consider alternate hypotheses and attempt to rule them out. For example, I can think of two hypotheses that might explain the data, but might not be as impactful as the one favored in the present version of the manuscript. The first is local in space - have the alkaline magmas simply inherited their S isotope compositions from local host rocks? I know the authors have the expertise to address this. The second is local in time - is the S source to the alkaline magmas simply roughly the same age as the magmas themselves? That is, instead of Gyr time lags in supply of surface S to the mantle source regions of alkaline magmas, can the data rule out 100 Myr time lags? In addition to these two hypotheses, I can also speculate a more starry-eyed one. The modelling illustrates an exquisite sensitivity of d34S values in these systems to f_O2 variations. Evidence for secular f_O2 variations in the mantle has been a 'holy grail' for Earth historians for a long time - can the d34S identified here be explained by temporal changes in f_O2 in the mantle source regions for alkaline magmas? From my non-expert perspective, one might be able to argue that small degree partial melts like alkaline magmas might be the right place to look for f_O2 variations rather that the historical focus on more extensive mantle melts like basalts and their siblings.

It is important to reiterate that the thrust of our paper is to establish whether alkaline rocks can be used to constrain magma source $\delta^{34}S$, and thus build a time-series of magma sources that goes significantly beyond the OIB record. In our revision we have added significant new $\delta^{34}S$ and S concentration data (Fig. 2, Table 1) which address our key hypothesis and demonstrate that magma sources can be reconstructed. For us this is the most important aspect of the paper and is the first time it has been done. Clearly, our compilation also throws up new questions about the causes of the secular change in igneous $\delta^{34}S$ (Fig. 6) and, as suggested by the reviewer, we now consider alternative hypotheses (lines 405-418 and in the new Figure 7) and attempt to rule them out (lines 420 – 438).

Our discussion focuses on the anomalous low-$\delta^{34}S$ Phanerozoic alkaline provinces, i.e. Kola and APIP. In brief, we are able to rule out the hypothesis that these data reflect crustal assimilation (i.e. are local in space) because the local crust is Precambrian, rather than Phanerozoic, and there is scant radiogenic isotope evidence for crustal assimilation (see references in lines 420–422). Moreover, we reject the hypothesis that low-$\delta^{34}S$ provinces are local in time (i.e. reflect 100 Ma time lags) because there is no evidence of Phanerozoic subduction zones in the vicinity of these provinces, ruling out subduction-related source modification (lines 424–428).

The reviewer's suggestion that temporal changes can be linked to source redox is difficult to assess because so few systems have both $\delta^{34}S$ values and robust estimates of mantle redox. In recent OIB literature Beaudry et al. 2018 (Nature Comms) identified a potential link between high oxidation state and high-$\delta^{34}S$ for the Canary Islands. This relationship is far from clear in our alkaline samples. For example, Ilímaussaq (one of our Greenland case studies) is one of the most reduced magmatic series known (fO_2 = QFM–4) and also shows positive $\delta^{34}S$ values (1–2 ‰). Without detailed work on mantle xenoliths and or melt inclusions it is not possible to assess this hypothesis and, ultimately, the simplest explanation is that our $\delta^{34}S$ time-series reflects $\delta^{34}S$ of the mantle source and hence hypotheses 2 and 3 (Figure 7) are the most likely.

Given that we are able to rule out signals that are local in space and time, our preferred hypothesis is that the low-$\delta^{34}S$ of Phanerozoic alkaline magmas reflect recycling via the deep mantle (lines 430–441). In our revision we have been clear that $\delta^{34}S$ alone is not unequivocal but that there is good evidence that both these provinces have a deep mantle origin, and in Kola (where high-quality Pb analyses are available) the source is suggested to be Archaean in age. We agree with the reviewer that $\Delta^{33}S$ would be a great addition but, carrying out this level of analysis for all systems was not feasible within the bounds of current time and resources between revisions.

A comprehensive understanding of the temporal trends in $\delta^{34}S$ (Fig. 6) will require significant further work (ideally $\Delta^{33}S$, but also radiogenic and stable isotopes for the same sample suite) and we have made this clear in lines 454–456. As the reviewer suggested we have improved the manuscript by posing and discussing alternate hypotheses and our choice of a deep crustal recycling scenario to explain the low-$\delta^{34}S$ provinces is based on solid reasoning. Our new $\delta^{34}S$ time-series will be of wide interest and will stimulate further geochemical studies to evaluate mantle evolution and test the new hypotheses we have posed here.

From a similar perspective, the current manuscript bases its speculation on something that is itself speculative. The hypothesis that there is a missing 34S-depleted reservoir from Earth's surface in the Proterozoic is a model-based inference. As far as I know it has not been verified with actual data, and the variability in the d34S record in pyrites through time looks like it might also support other hypotheses (for example, limited sulfate supply to the regions of pyrite formation).

The reviewer is correct that the original hypothesis of a missing ^{34}S-depleted reservoir was based on mass-balance modelling (Canfield, 2004, American Journal of Science). However, numerous studies have subsequently suggested that because the Archean and Proterozoic sedimentary rock record is biased toward positive $\Delta^{33}S$ and $\delta^{34}S$ that there is a S pool that is either under-sampled at the surface, deeply buried or lost to the mantle via subduction (Farquhar et al., 2010 Economic Geology).

The strongest evidence for the latter comes from the Mangaia and Pitcairn hotspots (Cabral et al., 2013, Nature and Delavault et al., 2016, PNAS) which recycle Archean crust with negative $\Delta^{33}S$ and $\delta^{34}S$ data. This hypothesis has been further developed by Farquhar and Jackson, 2016 (PNAS) and is supported by S isotopes studies of sedimentary basins which show that negative $\delta^{34}S$ is found in deepest settings (Shen et al., 2002, 2003, American journal of Science and Nature), which were most amenable to subduction.

We appreciate that not all hotspots show negative $\delta^{34}S$ (e.g. Samoa and Canary Islands) and that Beaudry et al., 2018 (Nature Comms) have linked very negative $\delta^{34}S$ to degassing processes. It has yet to be established whether Mangaia and Pitcairn experienced significant degassing, although it is worth noting that all samples analysed to date have negative $\delta^{34}S$ (< –2 ‰),values which are very similar to those required in Kola and APIP.

Ultimately, to explain the low-$\delta^{34}S$ provinces (Kola and APIP) requires a low-$\delta^{34}S$ source with a deep mantle noble gas signature. Since numerous studies (e.g. Farquhar et al., 2010 Economic Geology; Cabral et al., 2013, Nature; Delavault et al., 2016, PNAS; Farquhar and Jackson, 2016 PNAS and Giuliani et al. 2016 EPSL) have invoked an Archean and/or Proterozoic low-$\delta^{34}S$ reservoir to account for the negative $\delta^{34}S$ our decision to invoke a similar reservoir is consistent with current thinking.

In our revision we have emphasised that this is the currently accepted low-$\delta^{34}S$ source at several OIBs and have also highlighted these other studies (lines 443–445). Again, the focus of our manuscript is on developing the alkaline $\delta^{34}S$ record. While the precise cause for the secular variations is open to interpretation, we've done our best to put forward competing hypotheses and identify the origins of low-$\delta^{34}S$ provinces (as suggested by the reviewer, lines 420–450).

(3) Despite promises, this comment is about the first 4/5 of the current manuscript. The S isotopic modelling (and Fig 2 in particular) does not make it clear which results represent dependent variables, and which represent independent variables. My read is that the d34S values are measured independent variables, and the things like pH, f_O2, and T are all dependent variables. However this relationship is obscured by the choice to plot actual data in figure 2, with d34S values on the y-axis and pH, f_O2, and T on the x-axis. This is fine to do for model calculations, but gives the misleading impression that precise values for pH, f_O2, and T have been measured for each sample with a measured d34S value.

We have now separated out the data and model calculations into two plots (Figure 2 and Figure 4, respectively). Histograms (new Figure 2) provide a simple means of evaluating the $\delta^{34}S$ variations in our sample suite. Model-data comparisons (Figure 4) allow the reader to understand how changing temperature and fO_2 cause variations in $\delta^{34}S$, and how the data (shown as the coloured points) fit these models. To improve Figure 4 fixed model variables are now clearly labelled by a coloured box and the x-axis now reads 'Modelled fO_2' and 'Modelled temperature'. The latter, makes it clear that these are model calculations and we have emphasise in lines 219–235 that only samples with mineral pairs can be used to constrain temperatures.

Minor comment: the manuscript repeatedly refers to the "Precambrian" and to "Precambrian sulfur". More than almost any other geochemical quantity, however, Precambrian S is divisible between Archean and Proterozoic S. Making this distinction clear, I think, will go a long ways toward strengthening any interpretations made in a revised manuscript.

We have changed the wording and made this clear throughout.

Reviewer #3

1:

The authors describe how complex their system is: their highly evolved melts are granites. These are formed at low temperature, interact with fluids, and the authors have to invoke various pH of the fluids to derive the composition of the parental magma, noted δ34S∑S by the authors. Although the authors state that δ34S∑S is a mantle source estimate (L. 217), it is only a parental melt estimate.

Note this issue has been partly covered in our response to Reviewer 2 point (1).

The key goal of our study was to provide a detailed understanding of S isotope fractionation in alkaline systems and clearly the advantage of the Gardar is that a key parameters e.g. temperature, fO_2 and pH are well constrained. The reviewer is right to point out that at low

temperatures, particularly when other fluids are involved and the systems oxidise, then mineral $\delta^{34}S$ is a poor proxy for magma source $\delta^{34}S$.

In the original submission we focussed on the early-stage, high temperature, reduced alkaline intrusions. To strengthen our argument that these samples are representative of $\delta^{34}S$ in the parental magma and the mantle source we have added new data from magmatic cumulate and dykes, as well as primitive regional dykes and diatremes. The latter samples are accepted to be the most direct and deepest samples (lines 105–107, and commonly contain, small, but altered, mantle xenoliths).

All of these $\delta^{34}S$ values are positive and overlapping and in lines 292–296 we emphasise that: '…because the $\delta^{34}S$ source values calculated for early-stage sulphide-dominated rocks from the alkaline intrusions (Fig. 4a–c) are within ~1 ‰ of their parental magmatic units (Fig. 2), and also overlap the $\delta^{34}S$ of primitive samples; this provides strong evidence that evolved alkaline rocks constrain magma source $\delta^{34}S$.'

In our revised manuscript we also modelled a much wider array of magmatic processes that could have altered the primary mantle signature. As we outline below, these processes appear to have little impact on melt $\delta^{34}S$ (lines 165–217) and support the conclusion that alkaline rocks can be used to evaluate source $\delta^{34}S$.

From measured d34S values scattering between -1 and +4‰, the authors suggest parental melts compositions of 0, 2.0 and 2.5‰ for their three systems. Note that no uncertainty is offered for these estimates. These estimate relies on a isotope mass balance in the H2S-HS-S2- system in fluids at temperatures between 600 and 200 C. Discussion of how the parental melts could have acquired these three distinct d34S values is not offered in the detail. Instead, the authors assume that these values represent mantle sources – with no discussion offered, or citations given. Fractionating sulfides are known to occur during partial melting and early in the differentiation. The d34S effect is likely insignificant in basalts (No citation offered here) but unknown in lower-T melts.

The reviewer's comment breaks down into three key queries: 1) do our measurements reflect a mantle source; 2) can other processes have impacted melt $\delta^{34}S$ and 3) why have the parental melts acquired these enriched values.

We have addressed these as follows:

1) As noted above, in our revised manuscript we added new data from parental magmatic rocks, cumulate and primitive dykes and diatremes (Fig. 2 and Table 1). These new samples demonstrate that the $\delta^{34}S$ of evolved alkaline rocks are within ~1 ‰ of their parental magmatic rocks, and encompassed by the range of the most primitive rocks in the region (lines 292–300). Thus, all of our samples point toward an enriched mantle source and our case study suggests that magma source uncertainties are ±1 ‰ (although we assume larger uncertainties in our global compilation, Figures 5 and 6, lines 345–355)

The new $\delta^{34}S$ data from whole-rock samples at Ilímaussaq are a particularly useful addition because they confirm elevated $\delta^{34}S$ (1–2 ‰) in the magmas that are parental to the agpaitic rocks and veins (i.e. the sulphides in Fig. 2b). In our original submission we argued that the high pH of Ilímaussaq fluids (potentially ≥7) could explain the high-$\delta^{34}S$ of agpaitic rocks and veins even when $\delta^{34}S_{\Sigma S}$ was 0 ‰. In our revised manuscript we undertook new modelling to evaluate the role of pH and temperature separately (Figure 3c, lines 237–255). We found that pH models with $\delta^{34}S_{\Sigma S}$ fixed at 0 ‰ do not agree with the new whole-rock values $\delta^{34}S$, i.e. lines 248–250:

'[pH] models also predict that at high temperatures, $\delta^{34}S$ should converge to the bulk $\delta^{34}S_{\Sigma S}$ of 0 ‰, a feature not seen in our samples and contrary to Ilímaussaq whole-rock $\delta^{34}S$ which are mostly between 1 and 2 ‰ (Table 1).'

This supports temperature as the main control on early-stage mineral $\delta^{34}S$ and suggests an elevated source $\delta^{34}S$ at Ilímaussaq (1.8 ‰, Fig. 4a). Thus, all early-stage sulphide $\delta^{34}S$ measurements from the three complexes suggest similar source values of 1.8–2.5 ‰ (Fig. 4a–c). The reviewers' suggestions to provide $\delta^{34}S$ for parental magmatic rocks have really helped strengthen this case and we thank them for all their advice.

2) In our revised manuscript we evaluate S isotope fractionation due to crustal assimilation (Table 1, lines 173–189), magmatic degassing (Fig. 3a, lines 191–203), and sulphide segregation (Fig. 3b, lines 205–212) and show that these processes had minimal impacts on melt $\delta^{34}S$.

Focusing on fractionating sulphides, as suggested by the reviewer, we are able to demonstrate that because Gardar magmas are reduced (at or below QFM) then S in the melt is dominated by reduced species (S^{2-}, lines 192–197). Since S has the same valence in the melt and the sulphide (FeS) then isotopic fractionation is minor (Fig. 3b). Moreover, sulphide fractionation leads to negative $\delta^{34}S$ in the evolved melts, and cannot explain the overwhelmingly positive $\delta^{34}S$ observed in primitive magmatic rocks and early stages of the alkaline intrusions (lines 205–212).

3) In our revised manuscript we have provided new background information on the Gardar mantle source (lines 96–102). We explain that virtually all previous geochemical studies emphasise subduction zone metasomatism (~500 Ma before rift onset). Hence, 'Gardar magmas carry a geochemical signature of previously subducted crust and fluids[12–14]' and are likely to express this signature in their S isotope systematics.

In the discussion we have included a new section (Sulphur isotope signature of the Gardar magma source, lines 302–314) to underscore that our new results for an enriched mantle source with recycled surface S are coherent with these previous models.

2.

As said by the authors themselves, "d34S is exceptionally sensitive to low temperature oxidation" (L 217) but it also very sensitive to crustal assimilation. Granite-derived samples typically don't show significant S concentrations (< 10 ppm S). This is because sulfur was lost by sulfide segregation earlier in the differentiation sequence. The authors don't present the S contents of their melts, but the occurrence of any S is telltale of crustal assimilation or S inherited by crustal melting. Note that melts going through continental crust are well described to assimilate contaminants and drastically overprint the S isotope composition of the melts (Bekker et al. 2009 in science is one example among others – not cited)

We fully understand the reviewers concern that igneous samples with low S contents are susceptible to crustal contamination. In our revision we addressed this by including new analyses of $\delta^{34}S$ and S concentrations in our magmatic samples and in the local crust (new Table 1).

S concentrations in our alkaline rocks range from 100 to several 1000 ppm (Table 1) and are significantly greater than typical granitic magma series. S concentrations in the local crust are very low and when we consider various scenarios of crustal assimilation we find that even extreme crustal assimilation (50 %) have limited impact on melt $\delta^{34}S$ (generally <0.5 ‰, Table 1). Extreme crustal melting scenarios, modify $\delta^{34}S$ only slightly, and importantly are not supported by previous isotopic or petrographic studies from the Gardar (see references in line 187). Thus, we rule out significant crustal assimilation (lines 173–189).

It is also important to point out that only one of our ~100 $\delta^{34}S$ analyses was carried out on a Gardar granite (a granophyre dyke from Ivigtut) and so it is incorrect to refer to these samples as 'granites'. Alkaline rocks are among the most S-rich magma series known and we now make this clear in the introduction (lines 61–63), i.e. 'Carbonatites, for example, have average S concentrations of ~6000 ppm[15], much greater than other terrestrial magmas (granites and basaltic lavas typically have concentrations <100 ppm[16,17]).'

In fact, based on the data presented in this study, the authors cannot rule out the following suggestion: Melts that went through the Proterozoic crust are biased towards positive d34S because they have assimilated Proterozoic sediments (or contemporary S-bearing metasomatizing fluids) with positive d34S. Conversely, negative d34S observed elsewhere in phanerozoic settings are due to the assimilation of phanerozoic sediments (or phanerozoic S-bearing metasomatizing fluids) before emplacement of the melts. This is why the d34S record of alkaline melts mimics the d34S record of sediments.

These two scenarios are now summarised in the new Figure 7 and evaluated in lines 405–428. Our discussion focuses on Kola and APIP (the low-$\delta^{34}S$ alkaline provinces). In both settings Phanerozoic magmas intrude Proterozoic or Archean crust (ruling out assimilation of isotopically light Phanerozoic sediments), and there is no evidence of Phanerozoic suture zones (ruling out source modification by Phanerozoic S-bearing fluids or melts).

In our revised manuscript the reader will now fully appreciate why our favoured hypothesis is one of deep crustal recycling, i.e. because crustal contamination and SCLM modification can be ruled out, a mantle plume (which is also supported by noble gases from Kola and APIP) becomes the most likely candidate for the low-$\delta^{34}S$ magma source (430–441).

3.

Instead of discussing crustal assimilation, the authors assign their d34S estimate of parental melts to mantle sources. They present the idea that negative d34S values in phanerozoic granites are accounted for by the contribution of subducted components carrying negative d34S that have a Proterozoic age. Note that such components are not observed in the sedimentary record but have been invoked to be lost to the mantle by subduction to satisfy a surface mass balance

If the authors claim is correct, it seems to undermine their suggestion that d34S pattern in alkaline melts mimics the sedimentary record: The coinciding low d34S value in phanerozoic granites and sediments becomes fortuitous, since according to the authors themselves, granite values would be due to recycling of Proterozoic components with negative d34S, while sedimentary low d34S values are accounted for by the increase of sulfate content in the oceans after roughly 550 Ma.

As noted above we now include crustal contamination as one of our main hypotheses to explain the temporal trend in $\delta^{34}S$ (discussed in lines 405–428).

To explain the low-$\delta^{34}S$ provinces we require a low-$\delta^{34}S$ mantle source (see also our response to Reviewer 2's point 2). Since we rule out SCLM modification on ~10–100 Ma time-scales (hypothesis 2 in Figure 7, and lines 424–428), the only credible alternative is to invoke a mantle plume source for the anomalous low-$\delta^{34}S$. Again, a plume scenario is highly consistent with noble gas studies at Kola and APIP, and in the case of Kola, recent Pb-isotope work has suggested a recycled source of Archaean age (lines 430–435).

As the reviewer suggests, a low-$\delta^{34}S$ mantle reservoir of Proterozoic age is suggested by mass-balance (Canfield, 2004 American Journal of Science) and is also supported by evidence from sedimentary rocks which show negative $\delta^{34}S$ in deepest parts of Proterozoic

basins (e.g. Farquhar et al., 2010 Economic Geology; Shen et al., 2002, 2003, American journal of Science and Nature). Studies of mantle plume localities have subsequently invoked this low-δ^{34}S mantle pool to explain negative δ^{34}S and Δ^{33}S (Cabral et al., 2013, Nature and Delavault et al., 2016, PNAS) and have attributed this signature to a recycled Archaean source.

Given that numerous studies have appealed to a low-δ^{34}S recycled crustal source to explain their data at plume localities, our decision to invoke a similar reservoir is clearly in line with current thinking. We appreciate that in different plume settings (e.g. Samoa and the Canary Islands) other studies have invoked recycled Proterozoic sediments with positive δ^{34}S (~3 ‰). However, in the case of the Canary Islands the authors (Beaudry et al., 2018, Nature Comms) are uncertain about the age and composition of this material and suggested that serpentinized oceanic peridotites might also provide a viable source.

The goal of our manuscript is to show that alkaline rocks can be used to track mantle source and that their sources show both low- and high-δ^{34}S, akin to OIB. A full understanding of δ^{34}S in OIB and alkaline magmas will require (significant) further work and we would emphasise that the most exciting finding of our paper is that alkaline rocks suites allow us to understand crustal recycling over most of Earth history. In our revision we have significantly revised the discussion and have emphasised that there are uncertainties in OIB magma sources (lines 443–450) and the need for further work (lines 453–458).

We agree with the Reviewers second point (above) and have clarified that our preferred hypothesis means that the similarities in igneous and sedimentary δ^{34}S time-series (Figure 7) do not reflect connectivity on ~10–100 Ma time-scales, lines 435–437 :

[Our favoured hypothesis] 'implies that the co-variation of igneous and sedimentary δ^{34}S observed in Figure 6 is fortuitous since low-δ^{34}S provinces reflect recycling ancient (Ga) rather than contemporary (Ma) S.'

4.

Finally, I note that the authors have ignored studies that contradict their suggestion: The Samoa and Discovery hotspots (both of EM type) have d34S values consistent with the contribution of proterozoic sediments with positive d34S (+10‰) (Labidi et al. 2013, Nature – not cited and not shown in their fig. 4, and Labidi et al. 2015, EPSL – cited but not shown in their figure 4). Not only this contradicts the idea of negative d34S recycled in the mantle, it also shows that mantle storage of old components contributing to present-day volcanism does not lead to a d34S pattern that mimics the surface record. The authors claim of subduction of Proterozoic sediments with negative d34S is challenged by this literature.

Furthermore, one article by Beaudy et al. (2018, Nature Comm, not cited) shows that in fact, low d34S values in canaries magma are due to sulfur degassing. This hotspot is known to show recent recycled oceanic crust (Halliday et al. 1992,1993, Thirlwall, 1997). After reconstruction, Beaudry et al show that the mantle source of this hotspots include recycled components with positive d34S. This shows that even subduction of modern sediments do not meet the prediction made by Hutchison et al.

This literature seems to rule out the claims articulated by Hutchison et al.

Note that the Beaudry et al. (2018, Nature Comms) paper only came out when our manuscript was in review and in our original paper (Fig. 4) we chose only to present S isotope data for OIB samples with melt inclusions. We appreciate the reviewer's point that all this δ^{34}S literature should be included and have provided a detailed summary of this literature in the introduction (lines 33–43), and have also included this in Figure 6 (previously Figure 4).

In our revised manuscript we have now made it clear that similarity between igneous and sedimentary $\delta^{34}S$ records is coincidental, rather than being driven by a process that is local in space and time (lines 435–437). Indeed, several of the Phanerozoic alkaline complexes (e.g. Vulture and Yonghwa, Fig. 5) show high-$\delta^{34}S$ and so we have made it clear in the revised discussion that our focus is the anomalous low-$\delta^{34}S$ sources that are absent in Proterozoic (lines 405–407, 420–422 and 430–431).

As mentioned above (in response to the Reviewer's point 3) we appreciate other studies have invoked recycled Proterozoic sediments with positive $\delta^{34}S$ (~3 ‰). These studies are not definitive that all recycled Proterozoic sediments have positive $\delta^{34}S$ and indeed Beaudry et al. (2018, Nature Comms) suggested that serpentinized oceanic peridotites are also credible sources. We have now summarised this debate in the final section of the discussion (lines 443–450), and emphasise that the low-$\delta^{34}S$ provinces (Kola and APIP) require a low-$\delta^{34}S$ source with a deep mantle noble gas signature. As numerous studies (e.g. Farquhar et al., 2010 Economic Geology; Cabral et al., 2013, Nature; Delavault et al., 2016, PNAS; Farquhar and Jackson, 2016 PNAS and Giuliani et al. 2016 EPSL) have invoked an Archean and/or Proterozoic low-$\delta^{34}S$ reservoir to account for their low-$\delta^{34}S$ samples our decision to invoke a similar reservoir is in line with current thinking.

As noted in our response to Reviewer 2, it has yet to be established whether Mangaia and Pitcairn (the low-$\delta^{34}S$ OIB sources) experienced significant degassing, but it is worth noting that all samples analysed to date have negative $\delta^{34}S$ (< –2 ‰),values which are very similar to those required in Kola and APIP. In our revised manuscript we have clearly demonstrated that sulphur degassing has not impacted $\delta^{34}S$ in our Gardar samples (Figure 3b and lines 191–203). All alkaline samples compiled in our study (Fig. 5) contain abundant visible S phases (a requirement of mineral-scale $\delta^{34}S$ analysis) and undoubtedly have S contents comparable to the Gardar magmas (100's to 1000's of ppm). Therefore, it is highly unlikely that any of these alkaline magmas were significantly impacted by degassing.

We stress that the overarching goal of our manuscript was to establish an alkaline igneous $\delta^{34}S$ record and we are confident that we have achieved this. Our compilation clearly throws up new questions about the causes of the secular change in $\delta^{34}S$ and after evaluating various competing hypotheses in our revised manuscript we show that the low-$\delta^{34}S$ magmatic provinces require a low-$\delta^{34}S$ plume source. Various studies have suggested recycled ancient crust is a credible low-$\delta^{34}S$ source associated with plumes and we've clearly emphasised where this is consistent with current OIB literature (lines 443–450).

Reviewers' comments:

Reviewer #1 (Remarks to the Author):

I reviewed an earlier version of this manuscript and recommended the paper to be published after revision, taking into account my comments. The authors have done a thorough and excellent job with this revision, which addresses all the concerns I had. They clearly also carefully considered the comments by the other reviewers, some of which matched my own. This is an exciting and very interesting dataset documenting the evolution of global d34S through time. The authors do a good job presenting and defending their preferred scenario of deep recycling of Archaean crust. This dataset opens many avenues for more research into the global S cycle and I recommend publishing the new revised manuscript in its current form.

Cees-Jan De Hoog

University of Edinburgh

Reviewer #2 (Remarks to the Author):

I am satisfied by the authors' responses to the issues raised in my original review. With the exception of a very minor addition (a column of ages in the supplemental spreadsheet for the global compilation of d34S values from alkaline complexes), I think the current version of the manuscript is suitable for publication in Nature Communications.

Reviewer #3 (Remarks to the Author):

This is a significant improvement of the first version. The manuscript is easier to read and to follow. As a quick reminder, the authors argue that highly complex alkaline systems can be used to infer the d34S of OIB-derived parental melts. They use a case study of Greenland melts to make their point. They extrapolate their results to alkaline complexes worldwide. The authors speculate that a d34S secular evolution can be observed, assigned to evolution of the recycled crust.

Note that although the authors did not change their interpretation, it is now refined. In their last section, the authors now incorporate the idea of a time-lag between recycling and what comes out in magmas. They speculate that positive d34S observed for Proterozoic sediments is complemented by low d34S in sediments (that are not observed in the deep-time litterature). These would need to have been subducted and completely lost from the surface, and would later would show up in the mantle sources of some alkaline complexes.

This speculation relies on multiple assumptions and postulates, but is allowed. This is because the revised version now explicitely displays the implied assumptions (that were underlying/hidden in the previous version), which will benefit the reader.

For example, the authors have now explicitely incorporated a discussion on assimilation, which was lacking in a previous version. Whether their suggestion is correct or not (I may not be convinced), the current version offers the reader some literature on this critical issue. Note that Bekker et al (science 2008) remains uncited, although documenting a classic case of S-assimilation by melts emplaced in the continental crust.

A couple of key aspects may still be missing. For example, the authors have not discussed the distinctive trace element composition of sediments when it contributes to mantle sources (dented patterns, anomalies in Ce, anomalies in HFSE, etc). Therefore the varying d34S are not shown to be correlated with indexes of subducted components. In addition, the d34S response is evidently not systematic: some sources have a positive d34S, discussed by the authors as coming from Proterozoic sediments, in agreement with the work of Labidi et al (2013,2015). Some other sources have negative d34S (e.g. kola), also suggested as coming from Proterozoic sediments recycling. The observation of an evolving d34S, as well as prediction made by the authors, therefore both seem loose. However, I note that the authors clearly lay out explicitly two other possible hypotheses which, again, will provide the reader the required distance to the chosen interpretation.

Finally; I also note some lacking references. This is an easy fix. See my detailed, line-to-line comments below.

L. 39 – add Discovery hotspot, Labidi et al . 2013 (nature)

L. 60 – preserve mantle signature for trace element or major element does not imply a preservation of the mantle signature for volatiles like S

L 62 – carbonatities having 6000 ppm S could all be related to crustal assimilation, or exsolution of fluids from a silicate magma giving rise to a carbonate and S bearing magma. This may have nothing to do with a mantle source

L 63 – undegassed basaltic magmas have 1000-2000 ppm S, not < 100 ppm S. see mathez, 1976

L 65 – I would encourage the authors to consider saying that a S rich melt emplaced in a crustal environment can be produced by S assimilation, rather than asserting that a S rich magma has higher potential to study mantle sources.

L 67 – 73 : ok, good

L 168 – this assertion is not accurate. It represents the d34S of the fluid assuming there is no isotope fractionation between the phase and the fluid. I recommend the authors to find a supporting argument as to why there would be no fractionation

L 205 – ref 43 is Fiege et al. This reference is not addressing what the authors need. Only Labidi and Cartiny (2016), EPSL, characterized the fractionation associated with sulfide segregation. I recommend the authors to cite the correct references

This was suggested in the first round of review and remained ignored by the authors in both the revised manuscript and in the response letter. This does not reflect well on the revised study.

L 264 – I would recommend to reword, as in 'under equilibrium, bonding environment differences between oxidized and reduced species leads heavy isotope substitutions to favoured in sulphate', instead of 'sulphate favours 34S'. cite the review work by Schauble et al..

L 264, again: the equilibrium between sulfate and sulfide at 600 C is suggested to be roughly 8‰ by Miyoshi et al, 1984 (the only available experiments at high temperature for the sulfide sulfate isotope exchange => needs to be referenced here). the authors observe a d34S difference between

sulfate and sulfide that equals 10 to 15‰, larger than the equilibrium value (Fig 4c). This discrepancy could be reflecting assimilation of crustal sulfate, partially reduced to sulfide. This needs to be mentioned.

L 276 if a marine sulfate, with a d34S of +21‰, is partially reduced, the resulting d34S will increase above +21‰, precisely because 'under equilibrium, bonding environment differences between oxidized and reduced species leads heavy isotope substitutions to favoured in sulphate'. The figure shows increasing d34S along reduction, possibly reflecting the above comment. I recommend rewording the manuscript to reflect the figure (instead of, see L 278, 'barite at 21‰).

Again, here, the sulfides don't seem to ever be in equilibrium with the sulfates. This suggests a distinct origin and an explanation is warranted.

L 354-355 assuming that because the extrapolation to the parental melt at Gardar is valid does not allow to assume it will be valid elsewhere. This assertion is not substantiated by any data or logical development. In addition, it's unclear why the gardnar is within 1‰ of the parental melt, but other alkaline complexes are assigned to be within 1.5‰ of their parental melts.

L 445 – add Labidi et al (2013, Nature) for the discovery hotspot, with the original interpretation of a Proterozoic sediment causing positive d34S deviation in a mantle source containing recycled crust

Reviewer #1

No comments

Reviewer #2

I am satisfied by the authors' responses to the issues raised in my original review. With the exception of a very minor addition (a column of ages in the supplemental spreadsheet for the global compilation of d34S values from alkaline complexes), I think the current version of the manuscript is suitable for publication in Nature Communications.

Done

Reviewer #3

The authors have now explicitely incorporated a discussion on assimilation, which was lacking in a previous version. Whether their suggestion is correct or not (I may not be convinced), the current version offers the reader some literature on this critical issue. Note that Bekker et al (science 2008) remains uncited, although documenting a classic case of S-assimilation by melts emplaced in the continental crust.

We now cite Bekker et al. 2008 (Science) in line 73. We agree that this is a classic case of crustal assimilation impacting S isotopes; however, it is important to note that this paper is investigating komatiites. Komatiites have exceptionally high eruption temperatures (1400–1700 °C, Arndt and Nisbet, 1982, Komatiites), more than twice the value of erupting carbonatite melts (<600 °C, Jones et al., 2013, Reviews in Mineralogy & Geochemistry) and greatly exceeding the highest magmatic temperatures in our study area (~950 °C for Ilímaussaq nepheline syenites, Marks and Markl, 2015, Layered Intrusions). Komatiites represent high degree partial melts, and their extreme temperatures and magmatic fluxes are very much the opposite of the evolved alkaline magmas we have investigated.

In lines 60–62 we emphasise that alkaline rocks and carbonatites represent low degree partial melts, and that their low temperature, density and viscosity all favour rapid rise to the surface and reduce interactions with continental crust (this point has been made previously, e.g. Bell et al., 2002, EOS, cited). The vast majority of carbonatites preserve their mantle Nd and Sr isotope signatures, and indeed their Pb signatures are rarely contaminated by continental crust (even though carbonatite Pb concentrations are significantly lower than the crustal rocks). Recent review papers (e.g. Jones et al., 2013, now cited in Line 62) underscore this point in the opening paragraph: '[carbonatites] are derived from the mantle, showing almost no sign of contamination by the crust.'

In our previous revision we added significant new S concentration and isotopic data for our Greenland case study (40 $\delta^{34}S$ analyses and 37 S concentration analyses) and were able to rigorously assess crustal assimilation and rule it out (Table 1, lines 179–196). For the global comparison we provide key references for the anomalous S isotope provinces, e.g. Kola and Brazil, which use radiogenic isotopes to rule out crustal assimilation (lines 403–404, 443–445). In short, we have provided significant new data and references to the literature which all emphasise the limited role of crustal assimilation.

A couple of key aspects may still be missing. For example, the authors have not discussed the distinctive trace element composition of sediments when it contributes to mantle sources (dented patterns, anomalies in Ce, anomalies in HFSE, etc). Therefore the varying d34S are not shown to be correlated with indexes of subducted components. In addition, the d34S response is evidently not systematic: some sources have a positive d34S, discussed by the authors as coming from Proterozoic sediments, in agreement with the work of Labidi et al (2013,2015). Some other sources have negative d34S (e.g. kola), also suggested as coming from Proterozoic sediments recycling. The observation of an evolving d34S, as well as

prediction made by the authors, therefore both seem loose. However, I note that the authors clearly lay out explicitly two other possible hypotheses which, again, will provide the reader the required distance to the chosen interpretation.

Comparing the trace element signatures of different complexes to their $\delta^{34}S$ is a good idea and something we have considered. Although it is well established that alkaline rocks and carbonatites have similar trace element and isotopic signatures to OIB and recycled crustal sources (see new text and references in lines 53–54) it is quite difficult to compare trace elements patterns at different complexes. Unlike OIBs, different alkaline rocks have variable mineralogy, may have undergone variable fractionation and can also represent volcanic or plutonic assemblages. In basalts where one can simply analyse trace elements in the glass or whole-rock to evaluate melt composition the same is not true for alkaline rocks (due to the above issues). Where previous authors have compared trace elements at different complexes (e.g. Ingrid 1998, J. Pet.) it is often difficult to ascertain whether the pattern represents mantle source characteristics or mineral fractionation (e.g. Ce anomalies may reflect pyrochlore addition/removal). Trace element analysis of a single mineral phase, e.g. pyroxene, which is found at most complexes, would allow fractionation processes to be ruled out. However, such data do not exist in the literature and within the bounds of current time and resources it is not possible to add such a comprehensive new data set to our study and correlate $\delta^{34}S$ with trace elements.

It's important to remember that the goal of our study was to assess whether alkaline rocks provide a window into the deep S cycle. Our conclusion that S isotopes from many alkaline systems reveal a component of recycled surface S represents a significant advance and is consistent with many trace element studies. To address the reviewer's comment we have updated the text and added new references to pertinent trace element studies (e.g. lines 53–54 and 388–389), and have discussed trace element ratios in the Gardar and their implications for mantle source (lines 325–328).

We appreciate the reviewer's point that it's not possible to determine the precise age and source of the anomalous low-$\delta^{34}S$ magmatic provinces. In our manuscript we have been clear that $\delta^{34}S$ alone does not allow us to determine these source characteristics (e.g. lines 445–447 and 476–477) and it is worth underscoring that even for well-studied and well-characterised OIBs there is still much debate as to what causes the anomalous $\delta^{34}S$ in the mantle source. For example, at the Canary Islands hotspot recent S isotope investigations by Beaudry et al., 2018 (Nature Comms) are similarly unclear about the exact age and composition of the source (they consider Proterozoic sediments and serpentinized oceanic peridotites to be equally valid).

While we are confident that we have achieved the main aim of our study (i.e. to use alkaline rocks to constrain source $\delta^{34}S$ and build up a time-series of magma sources) our compilation inevitably throws up new questions about the origins of the low- and high-$\delta^{34}S$ magmatic systems. The reviewer acknowledges that we have done our best to interrogate a range of hypotheses and help the reader understand why our deep crustal recycling model is the best explanation for the data (Figure 7, lines 429–464). We've been clear about where our model is in agreement with other studies (lines 466–473), and highlighted the need for future work (lines 475–481). The reviewers all seem to agree that this is an important new data set and, quoting reviewer 1, 'opens many avenues for more research into the global S cycle'.

L. 39 – add Discovery hotspot, Labidi et al . 2013 (nature)

Done

L. 60 – preserve mantle signature for trace element or major element does not imply a preservation of the mantle signature for volatiles like S

We have removed 'preserve mantle signature' wording in lines 60–62 and make the simple and widely accepted point that alkaline magma are less susceptible to crustal contamination than other magmatic suites. Whether alkaline rocks can preserve a mantle S signature is discussed in lines 71–77. This now reads in a more logical manner.

L 62 – carbonatities having 6000 ppm S could all be related to crustal assimilation, or exsolution of fluids from a silicate magma giving rise to a carbonate and S bearing magma. This may have nothing to do with a mantle source

At virtually all carbonatites significant crustal assimilation can be ruled out (this point has been made above and in review papers, e.g. Jones et al., 2013, now cited in line 62).

The high concentration of S in alkaline rocks and carbonatites reflects the high solubility of S in these melts. Experimental studies on alkaline melts (Scaillet and Macdonald, 2006 J. Pet) demonstrate that a residual peralkaline melt (analogous to the evolved Gardar melts) will contain up to 1 % S, while experiments on carbonatitic liquids (Helz and Wyllie, 1979, GCA) also found similar values (eutectic melt containing 0.9 % S) . These authors underscore that S concentrations in alkaline melts are an order of magnitude higher than those of most silicate melts.

A key point made by these experimental studies is that evolved alkaline melts will retain most of their parental S. This is clearly expressed in the Scaillet and Macdonald, 2006 J. Pet paper which finds that peralkaline melts formed by fractional crystallisation of primitive alkali basalt will contain 60–90% of the initial S content of the parental magma. Thus, there is very good experimental evidence to show that evolved alkaline rocks retain much of the S from their primitive mafic sources.

This is also borne out by our data from the Gardar. At Ilímaussaq, when we can compare $\delta^{34}S$ of the early magmatic cumulates (averaging ~1.4 ‰) with latest stage melts (averaging ~1.8 ‰) there is virtually no difference (given typical analytical uncertainty is ±0.3 ‰).

To address this point we've changed the wording of this statement and cited these key references to emphasise that the most logical explanation for the high S contents is S solubility (lines 62–64).

L 63 – undegassed basaltic magmas have 1000-2000 ppm S, not < 100 ppm S. see mathez, 1976

We have confirmed that we are referring to terrestrial lavas (i.e. those erupted on the continents) which are mostly degassed. Undegassed magmas are predominantly erupted in the oceans and won't be preserved beyond ~200 Ma.

Line 65 now reads: 'Carbonatites, for example, have average S concentrations of ~6000 ppm[28], much greater than other terrestrial magmas erupted through continental crust (granites and degassed basaltic lavas typically have concentrations <100 ppm[1,29]).'

L 65 – I would encourage the authors to consider saying that a S rich melt emplaced in a crustal environment can be produced by S assimilation, rather than asserting that a S rich magma has higher potential to study mantle sources.

We've added the uncertainty to this statement, line 68–69: 'they are potentially well suited for understanding S cycling between the surface and mantle.'

The specific complexities of using S-rich alkaline melts to evaluate mantle sources (e.g. crustal contamination) are then discussed in the following paragraph (lines 71–77).

L 67 – 73 : ok, good

Now lines 71–77, we feel that this section is addressing the reviewers point above

L 168 – this assertion is not accurate. It represents the d34S of the fluid assuming there is no isotope fractionation between the phase and the fluid. I recommend the authors to find a supporting argument as to why there would be no fractionation

Our previous wording was unclear and we've updated this, lines 171–174:

'Mineral $\delta^{34}S$ records the isotopic fractionation between the S mineral phase and the melt or fluid. For an individual S-bearing mineral, the measured $\delta^{34}S$ reflects the $\delta^{34}S_{\Sigma S}$ but also the temperature and S speciation of the melt/fluid (the latter being controlled by pH and fO_2 conditions[36]).'

L 205 – ref 43 is Fiege et al. This reference is not addressing what the authors need. Only Labidi and Cartiny (2016), EPSL, characterized the fractionation associated with sulfide segregation. I recommend the authors to cite the correct references

We have added this reference and kept the reference to Fiege et al. because this paper describes the fractionation factors used in Fig. 3b. Lines 212–215 now read:

'Although measurements of $\delta^{34}S$ in mafic rocks suggest negligible isotopic fractionation between melt and sulphide mineral phases[52] we decided to evaluate sulphide (FeS) segregation at fO_2 conditions relevant to the Gardar (using equations of ref. [35] and fractionation factors for silicate melts[51], Fig. 3b).'

L 264 – I would recommend to reword, as in 'under equilibrium, bonding environment differences between oxidized and reduced species leads heavy isotope substitutions to favoured in sulphate', instead of 'sulphate favours 34S'. cite the review work by Schauble et al..

Done, lines 265–268 : 'At equilibrium, differences in bond stiffness between oxidised and reduced S species favour heavy ^{34}S isotope substitutions in sulphate[58] and lead to a sharp $\delta^{34}S$ decrease in co-existing sulphides.'

L 264, again: the equilibrium between sulfate and sulfide at 600 C is suggested to be roughly 8‰ by Miyoshi et al, 1984 (the only available experiments at high temperature for the sulfide sulfate isotope exchange => needs to be referenced here). the authors observe a d34S difference between sulfate and sulfide that equals 10 to 15‰, larger than the equilibrium value (Fig 4c). This discrepancy could be reflecting assimilation of crustal sulfate, partially reduced to sulfide. This needs to be mentioned.

The temperatures of these late-stage units (i.e. the data shown in the right hand panel of Fig. 4a–c and all of Fig. 4d) is <600 °C. These deposits represent the final stage of the magmatic fluids and we have flagged their low temperature in the Geological setting (lines 129–134) and in Figure 4, where the coloured boxes show fixed modelling parameters (including temperature).

Using the fractionation factors compiled by Seal 2006 (Reviews in Mineralogy & Geochemistry, cited), temperatures of 200–300 °C readily account for the observed differences in sulphate-sulphide $\delta^{34}S$. There is also no evidence for sulphate deposits in any of the Gardar crustal rocks.

To address the reviewers concerns we have added a sentence to our description of S in the local crust (lines 181–183):

‘Eriksfjord sediments have high-$\delta^{34}S$ (25 ‰, consistent with a marine origin) but minimal S concentrations (~10 ppm) and we stress that there is no evidence for evaporitic units or shales with high S contents.’

We also include these key references (Seal 2006 and Miyoshi et al., 1984) for sulphate-sulphide equilibrium values and implications for temperatures in lines 266–269:

‘Unlike the early-stage samples, late-stage veins and fenites (Fig. 2c,e,g) contain sulphates and sulphides. The difference between sulphate and sulphide $\delta^{34}S$ is up to 15–25 ‰ in these late-stage samples (Fig. 4a–d) and suggests low temperatures of formation 200–300 °C (for reference isotope fractionation at magmatic temperatures, ~600 °C, is ~8 ‰[50]).’

L 276 if a marine sulfate, with a d34S of +21‰, is partially reduced, the resulting d34S will increase above +21‰, precisely because ‘under equilibrium, bonding environment differences between oxidized and reduced species leads heavy isotope substitutions to favoured in sulphate’. The figure shows increasing d34S along reduction, possibly reflecting the above comment. I recommend rewording the manuscript to reflect the figure (instead of, see L 278, ‘barite at 21‰).
Again, here, the sulfides don’t seem to ever be in equilibrium with the sulfates. This suggests a distinct origin and an explanation is warranted.

We have reworded this sentence as suggested, lines 288–292:

‘As noted above, differences in bond stiffness between oxidised and reduced S species favour heavy isotope substitutions in the sulphate[58] (Fig. 4d) and so our model rationalises the presence of barite with $\delta^{34}S$ up to 22.5 ‰ as well as the pyrite and galena with elevated and wide-ranging $\delta^{34}S$ (since small variations in redox lead to large isotopic shifts, 3–10 ‰, Fig. 4d)’

Regarding the reviewer’s comment about isotopic equilibrium between the sulphates and sulphides in the Fig. 4d it is important to note that:

1) These units represent the final stages of the magmatic deposit and so must have formed at temperatures <300 °C (i.e. lower than all temperatures suggested by the S mineral pairs in Fig. 4c)

2) There is strong evidence from fluid inclusion studies (Kohler et al., 2009, Lithos, cited) that these final assemblages record the influx of a meteoric source with a isotopic and chemical composition analogous to a Canadian Shield Brine (detailed in the Supplementary information, lines 198–204)

3) Although the sulphates and sulphides are found in the same deposits it is difficult to pull out pairs that are in direct contact

To model the isotopes in these samples we used an equilibrium fractionation model. While this is undoubtedly an oversimplification of how an external brine interacts with a reducing magmatic system it provides it a good generalized model for evaluating the isotopic evolution of a Canadian Shield brine (above) as it is reduced.

The advantage of this model is that we were able to test the predictions made by earlier fluid inclusion studies and use independent constraints on the pH and $\delta^{34}S_{\Sigma S}$ (typical of these external brines, see references cited in Supplementary Information lines 232–235. This model accounts for the co-precipitation and correct isotopic values of the sulphides and sulphates, and we also note that the required redox conditions (between QFM and QFM+2)

are very similar to those suggested for the opposite processes (i.e. oxidation of the magmatic fluid in Fig. 4c).

Given that temperatures in these deposits are low and there is no evidence of local S-rich rocks to assimilate (see comment above) we are convinced that this provides the simplest and only explanation for the observed isotopic values (and crucially, it is in full agreement with earlier fluid inclusion studies). The model in Fig. 4d clearly shows that small changes in redox will lead to significant isotopic shifts and without analysing large numbers of samples it is very difficult to constrain the precise isotopic fractionation between sulphides and sulphates in these deposits. And hence, these late-stage assemblages are never in the magma source reconstruction (lines 369–376).

To address the reviewers query we have modified lines 292–297: ‘Although these final-stage sulphates and sulphides are rarely found in direct contact (and do not provide unequivocal evidence for equilibrium), our model complies with previous evidence for late-stage brine influx (e.g. fluid inclusions[14]), and strengthens the case that Ivigtût represents a heterogeneous mixing zone between a reduced CO_3^{2-} and F^- rich magmatic fluid ($\delta^{34}S_{\Sigma S}$ = 2.5 ‰) and oxidized brine ($\delta^{34}S_{\Sigma S}$ = 20 ‰).’

L 354-355 assuming that because the extrapolation to the parental melt at Gardar is valid does not allow to assume it will be valid elsewhere. This assertion is not substantiated by any data or logical development. In addition, it’s unclear why the gardnar is within 1‰ of the parental melt, but other alkaline complexes are assigned to be within 1.5‰ of their parental melts.

In our revision we have clarified our logic on why it is appropriate to apply these methods globally (with large but realistic uncertainties). The first point is that our Gardar case study analysed a broad suite of alkaline rocks, lines 364–366:

‘…our detailed case study demonstrates that a wide variety of alkaline rocks, including magmatic cumulate, late-stage silicate melts, carbonatites and aluminofluoride melts (Ivigtût), closely approximate source $\delta^{34}S$ (within ~1 ‰).’

We specifically analysed silicate melts from a wide compositional range (primitive mafic dykes and lavas through to highly-evolved agpaitic residual melts), as well as carbonatites and unusual carbonate and fluorine-rich melts from Ivigtût. These melts are characteristic of alkaline systems globally and the fact that they all show limited fractionation in their early sulphide-dominated assemblages provides robust evidence that we can use all of these rock types to evaluate magma source $\delta^{34}S$.

The second point is that our global compilation (Figure 5, discussed in lines 339–359) clearly demonstrates that the chemical evolution of Gardar alkaline melts is not unique and that similar isotopic shifts can be identified in many complexes, lines 366–379:

‘Given that virtually all alkaline systems mirror the $\delta^{34}S$ trends observed in the Gardar (Fig. 5) it is reasonable to assume that the isotope systematics that govern Gardar melts are applicable elsewhere.’

This is good evidence that the processes that govern melt, fluid and sulphur isotope evolution are comparable at all alkaline systems, and thus it is very reasonable to assume that the lessons from one system apply to others.

We have also added a sentence to clarify the logic on our source uncertainty, lines 376–379:

‘Based on our case study, where >95 % of all early-formed S minerals (excluding galena, above) are within ±1.5 ‰ of the most primitive $\delta^{34}S$ values, we propose similar large but reasonable uncertainties on our magma source $\delta^{34}S$ (Fig. 5).’

The reviewers have made it clear that we have been honest about our assumptions throughout the manuscript. In our revision we have also stressed that while we are using

early-stage sulphides to evaluate magma source $\delta^{34}S$ '…primitive alkaline magmas are undoubtedly the best method' (lines 363–364). Given that is not feasible to repeat our comprehensive analysis of $\delta^{34}S$ in primitive melts for every alkaline system, the reader should be able to appreciate that our approach works exceptionally for a wide variety of alkaline magmas, that many of these complexes show similarities in their isotope systematics and that our estimates of source uncertainty are realistic and well-constrained by our new observations.

L 445 – add Labidi et al (2013, Nature) for the discovery hotspot, with the original interpretation of a Proterozoic sediment causing positive d34S deviation in a mantle source containing recycled crust

Done

REVIEWERS' COMMENTS:

Reviewer #3 (Remarks to the Author):

the authors have made commendable efforts and the manuscript is now excellent. I am looking forward to read this paper in Nature Communication.
